# Monitoring HIV Antiretroviral Therapy via Aptamer-Based Measurements in Preclinical Animal Models, in Human Plasma

**DOI:** 10.1002/adsr.202400191

**Published:** 2025-01-07

**Authors:** Jing Li, Vincent Clark, Chen-Hsu Yu, Karen Scida, Miguel Aller Pellitero, Rolando L. Albarracín Rivera, Wenrui Zhong, Erin Demek, Jeffrey Fountain, J.D. Mahlum, Richard E. Haaland, Gregory V. Carr, Jonathan Sczepanski, Netzahualcóyotl Arroyo-Currás

**Affiliations:** Texas A&M University, College Station, TX 77843, USA; Johns Hopkins University, Baltimore, MD 21218, USA; Texas A&M University, College Station, TX 77843, USA; Lieber Institute for Brain Development, Johns Hopkins University School of Medicine, Baltimore, MD21205, USA; Department of Pharmacology and Molecular Sciences, Johns Hopkins University School of Medicine, Baltimore, MD21205, USA; Texas A&M University, College Station, TX 77843, USA; Texas A&M University, College Station, TX 77843, USA; Johns Hopkins University, Baltimore, MD 21218, USA; Division of HIV Prevention, Centers forDiseaseControl and Prevention, Atlanta, GA 30329, USA; Johns Hopkins University, Baltimore, MD 21218, USA; Division of HIV Prevention, Centers forDiseaseControl and Prevention, Atlanta, GA 30329, USA; Lieber Institute for Brain Development, Johns Hopkins University School of Medicine, Baltimore, MD21205, USA; Department of Pharmacology and Molecular Sciences, Johns Hopkins University School of Medicine, Baltimore, MD21205, USA; Texas A&M University, College Station, TX 77843, USA; Johns Hopkins University, Baltimore, MD 21218, USA; Department of Pharmacology and Molecular Sciences, Johns Hopkins University School of Medicine, Baltimore, MD21205, USA

**Keywords:** antiretroviral therapy, aptamer, biosensor, HIV

## Abstract

Monitoring the concentration of antiretroviral drugs is critical to ensuring patient adherence to HIV treatment and prevention regimens, which is crucial for drug efficacy. These tools may also be useful for screening samples at blood donation centers to avert potential new infections. The current benchmark method to support antiretroviral drug monitoring, liquid chromatography coupled to mass spectrometry (LC-MS), requires centralized facilities with costly instrumentation, and has blood-to-result turnaround times of days to weeks, making it impractical for effective drug monitoring. Seeking to overcome this issue, an aptamer is developed for the antiretroviral drug emtricitabine, which is present in most antiretroviral combination therapies in the market and used for both infection management and prevention. The aptamer has clinically relevant sensitivity in biofluids and is highly selective relative to close analogs such as cytosine, cytidine, fluorocytidine, and lamivudine (a.k.a. 3TC). Using this aptamer, two analytical assays are developed, one for continuous, in-vivo emtricitabine monitoring in rodent research models, and one for rapid and high-throughput screening of emtricitabine levels in human plasma. Through blinded analytical validation, this clinical assay achieved an 86.9% positive and 100% negative correlation, with an overall agreement rate of 95% relative to the benchmark LC-MS method.

## 1. Introduction

**Figure 1.**
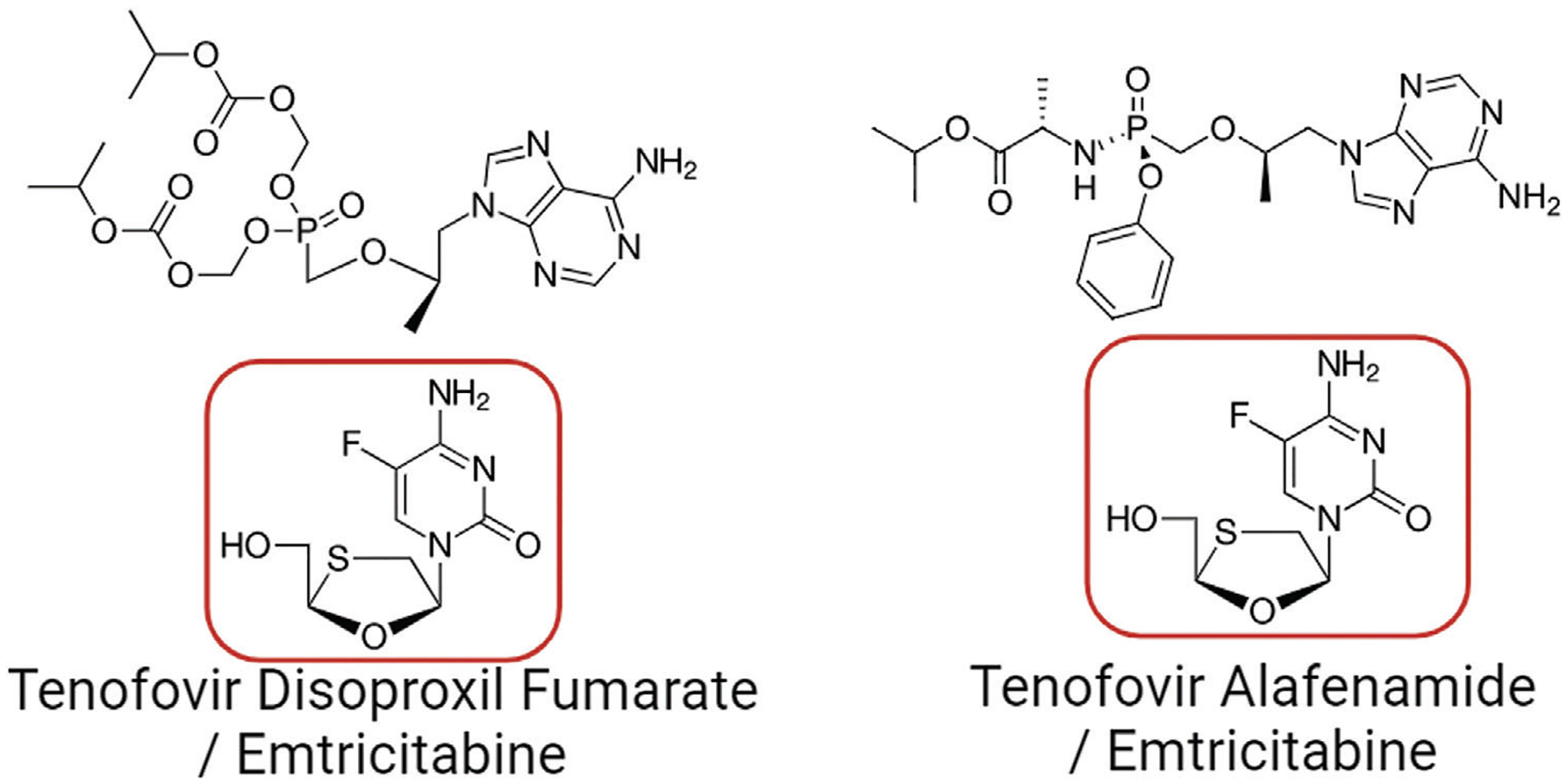
The therapeutic emtricitabine (FTC, circled in red) is found in two of the most used antiretroviral (ARV) therapies for HIV treatment and prevention in tandem with tenofovir disoproxil fumarate (top left) or tenofovir alafenamide (top right), making FTC an ideal target for drug adherence studies.

Human immunodeficiency virus (HIV) infection continues to be a global public health concern with an estimated 39 million people living with HIV worldwide and 1.5 million new infections each year.^[1]^ Antiretroviral (ARV) drug therapy has been identified as a key pillar to end the HIV epidemic in the United States.^[2]^ Daily oral dosing of ARV combinations that directly inhibit HIV replication allows people living with HIV (PLWH) to maintain suppressed viral loads, improve clinical outcomes, hinder the emergence of drug-resistant virus, and prevent subsequent transmission to uninfected individuals.^[3,4]^ Additionally, daily oral dosing with the ARVs tenofovir disoproxil fumarate (TDF) or tenofovir alafenamide (TAF) in combination with emtricitabine (FTC, Figure 1) is highly effective at preventing HIV infection amongst healthy men and women.^[3,4]^ However, ARV effectiveness in HIV treatment and prevention is tightly associated with adherence to daily oral dosing. In response, long-acting injectable ARV formulations have been developed to reduce reliance on daily regimens.^[5]^ Nonetheless, irrespective of the dosing schedule, adherence to ARV therapy remains critical to sustaining viral suppression and preventing infection.

Because adherence to HIV treatment is so critical to therapeutic efficacy, monitoring ARV concentrations in biofluids is necessary for the development and implementation of HIV treatment and prevention strategies. Preclinical and clinical evaluation of ARVs and novel dosing strategies requires pharmacokinetic assessment to define subject-specific concentrations associated with viral load suppression or protection from infection.^[6,7]^ ARV concentration monitoring is increasingly being considered for objective measures of adherence to HIV treatment and for the establishment of prevention strategies.^[8]^ This contrasts with previously used self-reporting methods which were unreliable.^[9]^ Additionally, screening samples for ARVs is critical to the identification of unreported ARV use in cases of people misreporting their HIV status during behavioral surveillance studies and at blood donation centers.^[10]^ Thus, there exists a growing critical need to support ARV monitoring at the point of care and specimen collection venues at a larger scale than currently being conducted.

This study reports the development of a nucleic acid aptamer binding to the ARV drug, FTC, which is a component of the standard-of-care combination therapy to both treat HIV and prevent infection (prophylaxis). The aptamer is completely selective against close analogs of FTC, including cytosine, cytidine, deoxycytidine, fluorocytidine, and lamivudine (a.k.a. 3TC). We integrated this aptamer into two sensing modalities: an electrochemical approach that allows real-time monitoring of FTC pharmacokinetics in live rodents for biomedical research applications, and an optical approach that enables, for the first time for aptamers in high-throughput format, the monitoring of ARV concentrations in patient plasma. In a blinded comparative validation against the current benchmark LC-MS method, our optical diagnostic assay achieves 100% correlation in negative samples and >95% correlation with positive samples, paving the way for high-throughput ARV monitoring.

## 2. Results and Discussion

Nucleic acid aptamers are synthetic biorecognition elements that bind molecular targets with high specificity. Aptamers that bind small molecules often have affinities in the tens of nanomolar^[11,12]^ to a few micromolar range.^[13]^ Their affinity is limited by the low surface area of small molecule targets and the few functional groups they typically display. However, aptamers are ideal for ARV monitoring because these drugs are administered at high doses that result in pharmacokinetic profiles with hundred nanomolar to micromolar concentrations in blood.^[14]^ Nucleic acids can be easily modified to be nuclease-resistant and functionalized with reporters to support signal transduction.^[15]^ Here, we specifically designed conformation-switching aptamers that either transfer electrons at a faster rate or emit fluorescence, when in the presence of FTC, enabling preclinical and clinical measurements of FTC concentrations in unprocessed biofluids in vitro and in vivo.

**Figure 2.**
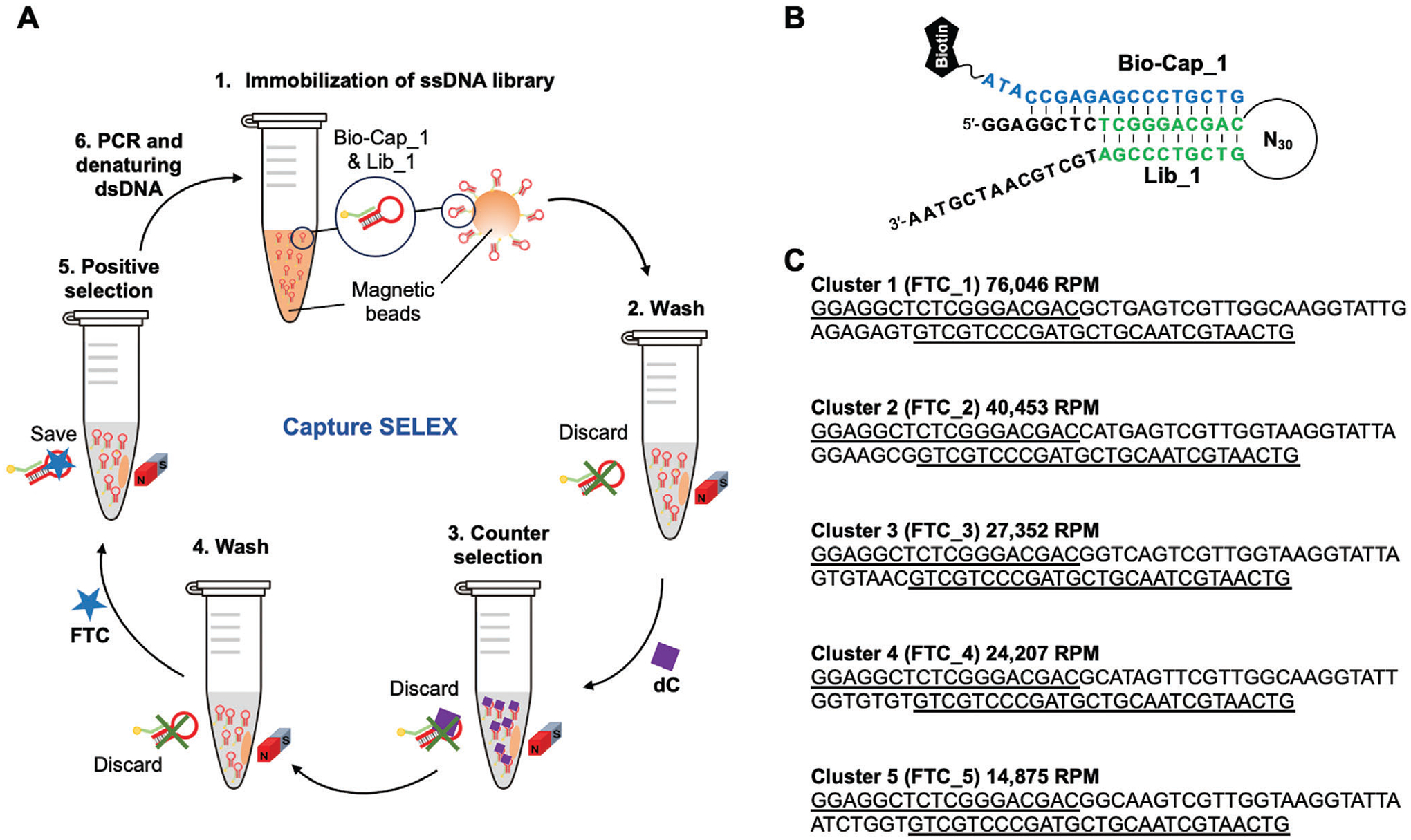
Selection of DNA aptamers against FTC. A) The capture-SELEX procedure: 1) The ssDNA library (Lib_1) is immobilized to streptavidin-coated magnetic beads via a biotinylated capture strand (Bio-Cap_1); 2) The beads are washed to remove weakly bound ssDNA; 3) Counter selections are carried out against deoxycytidine (dC, from round 9 onward); 4) The beads are washed to remove unbound ssDNA from the counter selection; 5) The positive selection step is carried out in the presence of FTC; 6) ssDNA eluted during the positive selection step is amplified by PCR and made single stranded for the next SELEX round. B) Sequence of Bio-Cap_1 and sequence and secondary structure of Lib_1. C) Parent sequences for the top-5 clusters identified after 11 rounds of capture-SELEX. Sequences and structures of all oligonucleotides used in this work are listed in Table S1 (Supporting Information). Underscored text indicates conserved primer regions.

To isolate aptamers with selective binding affinity for FTC, we employed a modified version of capture-SELEX (**S**ystematic **E**volution of **L**igands by **Ex**ponential Enrichment)^[16]^ (Figure 2A). The single-stranded DNA (ssDNA) library (Lib_1) contained a 30-nucleotide random domain flanked on both sides by fixed sequences with partial complementarity, resulting in the formation of a stem-loop structure (Figure 2B). To begin the selection, the library (≈10^14^ individual molecules) was immobilized onto streptavidin-coated magnetic beads via a biotinylated capture strand (Bio-Cap_1 in Figure 2B) that also disrupts the formation of the stem-loop.^[17]^ After extensive washing to remove unbound sequences, the immobilized library was exposed to 100 μm FTC in PBS buffer (*p*H = 7.2) containing 2 mm Mg^2+^ (i.e., the positive selection step). Aptamer sequences that bound FTC and consequently underwent a conformational change caused by stabilization of the stem-loop were spontaneously released from the capture strand on the beads and collected for PCR amplification. The double-stranded DNA (dsDNA) resulting from PCR amplification was used to generate the corresponding pool of ssDNA for the next selection round.

The selection pressure was gradually increased during successive SELEX rounds by decreasing the concentration of FTC and the duration of the positive selection step. Supplementary Table S2 (Supporting Information) summarizes the selection conditions for each round. In rounds 9 and onward, we incorporated a counter-selection step wherein the immobilized DNA library was first exposed to deoxycytidine (dC), which is structurally similar to FTC. Aptamer sequences that bound dC were displaced from the beads and discarded; aptamer sequences that remained immobilized were advanced into the positive selection step against FTC. The progress of the selection was monitored by comparing the amount of DNA eluted by FTC relative to the amount eluted during the washing and counter-selection steps (Figure S1, Supporting Information). After 11 rounds, this analysis revealed that more DNA was being eluted by FTC (Figure S2, Supporting Information) compared to the washing steps and in much less time compared to the counter-target dC, and thus, the selection was halted at this point.

**Figure 3.**
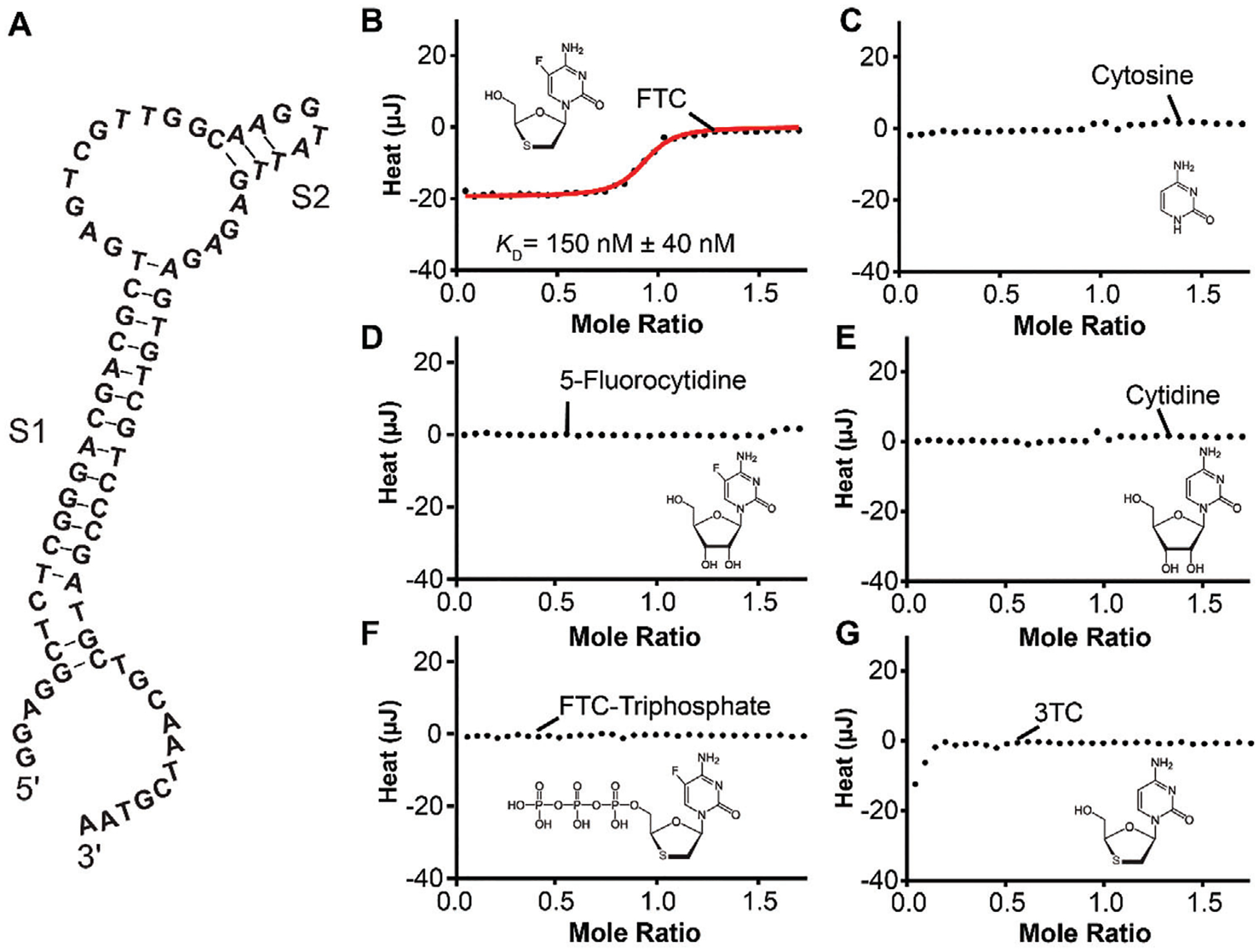
Characterization of FTC_1_Full binding affinity and selectivity. A) Sequence and Nupack-predicted^[20]^ secondary structure of FTC_1_Full. B) FTC affinity measurement via isothermal titration calorimetry (ITC) displays a dissociation constant of 150 nm ± 40 nm. Challenging the FTC_1_Full sequence with C) cytosine, D) 5-fluorocytidine, E) cytidine, or F) FTC-triphosphate resulted in no heat exchange, underscoring the high selectivity of this aptamer for FTC relative to close structural analogs. G) Upon challenging the sequence with lamivudine (a.k.a. 3TC), a low heat exchange is observed early in the titration, which may be indicative of a non-specific interaction with the aptamer. The solid circles in all thermograms represent the data from N = 1 measurements. The red line in panel B represents a non-linear regression to the Hill Isotherm, with Hill coefficient = 1.

**Figure 4.**
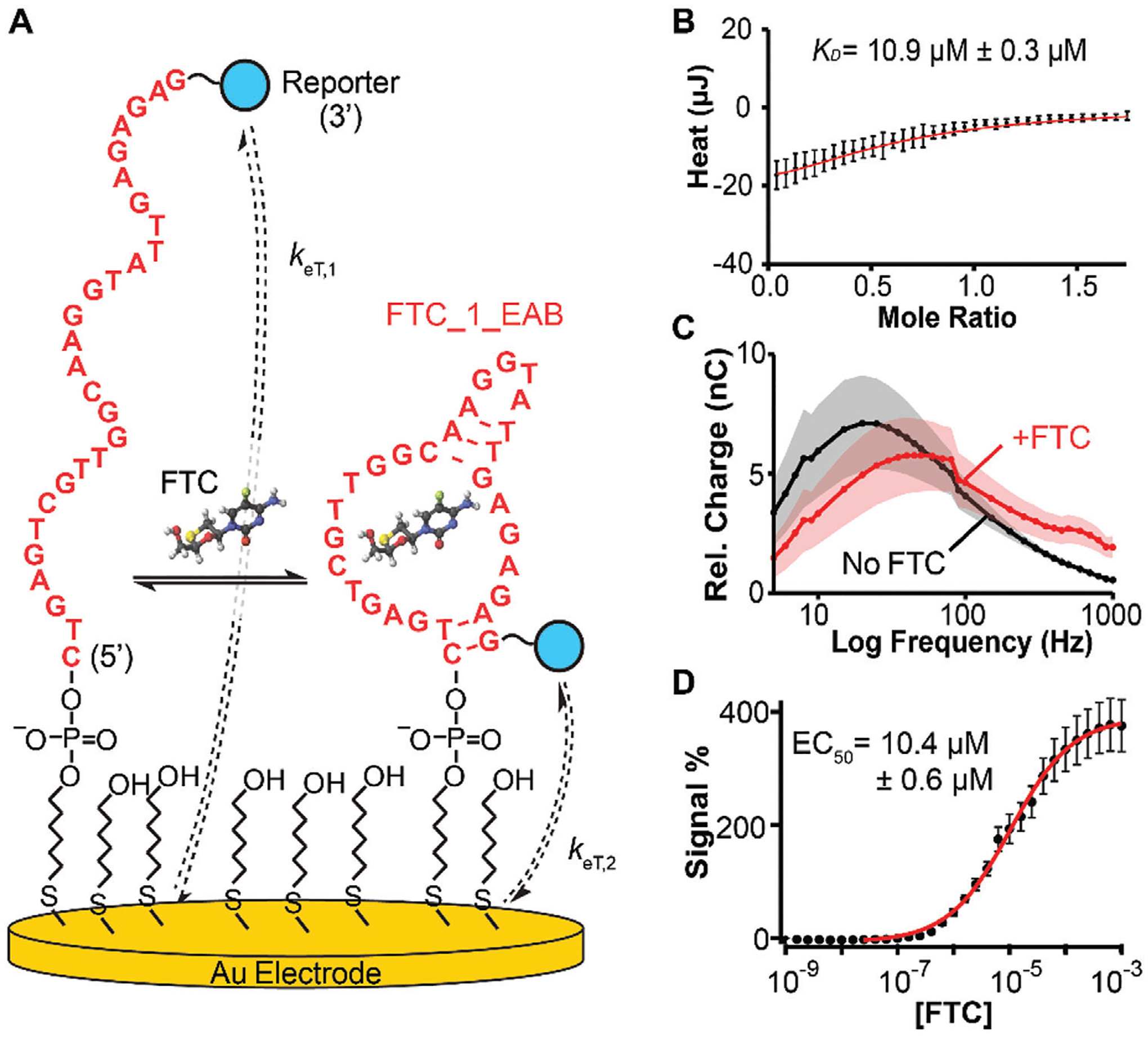
Development of electrochemical, aptamer-based (E-AB) sensors for FTC. A) The E-AB sensing mechanism leverages changes in electron transfer rates (*k*_eT,1_ vs *k*_eT,2_) driven by target binding. In the bound state, the aptamer is favored to form a secondary structure that places the redox reporter (blue circle) closer to the electrode surface, increasing electron transfer kinetics, *k*_eT,2_ >> *k*_eT,1_. To enable this mechanism, we truncated FTC_1_Full from 72 to 28 nt to destabilize stem S1, resulting in sequence FTC_1_EAB. B) This truncation retained FTC binding as determined via ITC measurements, albeit with a loss in affinity. These thermograms represent the mean of five measurements. Errors indicate the standard deviation. (C) The FTC_1_EAB sequence was modified with hexanethiol at the 5′ ends and methylene blue (reporter) at the 3′ ends and immobilized on gold electrodes as shown schematically in panel A. Interrogating this modified aptamer in the absence (black) and presence (red) of 10 μm FTC via square wave voltammetry resulted in frequency maps that shifted maxima to higher frequencies (i.e., higher electron transfer rate) in the presence of FTC. Solid circles and connecting lines represent the average of 8 individual electrodes. Shaded regions illustrate their standard deviation. D) Challenging FTC_1_EAB with increasing concentration of FTC in phosphate-buffered saline produced dose-response curves covering the clinical range for FTC. The solid circles represent the average from 8 individual electrodes. Errors illustrate the standard deviation. The red line shows a non-linear regression to the Hill isotherm, using the indicated EC_50_ and a Hill coefficient = 1.

Candidate aptamers from the eleventh round of selection were identified using next-generation sequencing (NGS) and bioinformatics analysis using the FASTAptamer toolkit.^[18]^ The 4620 unique sequences identified by this analysis were clustered using a Levenshtein edit distance of 4 and the clusters ranked based on the abundance of the parent sequence (Figure S3, Supporting Information). Parent sequences from the top five clusters (Figure 2C) were synthesized and screened for affinity (Figure S4, Supporting Information). The parent sequence from cluster 1 (Figure 3A) showed an affinity for FTC in the few hundred nM range (Figure 3B) and was coincidentally the most abundant sequence in the enriched pool. Additionally, this sequence underwent target-induced conformation switching as evaluated via electrochemistry (Figure 4C). This sequence, here named FTC_1_Full (see Table S1, Supporting Information), was carried forward for further biophysical characterizations (Figure 3).

We characterized the binding affinity of FTC_1_Full against FTC using isothermal titration calorimetry (ITC) [see Methods and reference^[19]^]. For these measurements, we employed the aptamer sequence as the receptor (at a concentration of 20 μm) and titrated FTC from a stock solution (at 200 μm). The resulting ITC thermogram (Figure 3B) revealed a dissociation constant of 150 nm ± 40 nm, which matches clinically relevant concentrations of FTC (> 160 nm at steady-state dosing).^[14]^ Next, we investigated the selectivity of the aptamer against five structurally related compounds, cytosine (Figure 3C), 5-fluorocytidine (Figure 3D), cytidine (Figure 3E), FTC-triphosphate (Figure 3F), and lamivudine (Figure 3G). Remarkably, FTC_1_Full displayed negligible heat exchange in the presence of the structural analogs. These data suggest that FTC_1_Full interacts with the FTC nucleoside through both its base and sugar moieties, and strongly discriminates based on the presence of the 5-fluoro substituent as seen for lamivudine (a.k.a. 3TC). Given this specificity, we continued developing the aptamer for diagnostic assays.

We set out to develop electrochemical and fluorescence-based sensors for two independent FTC measurement applications by exploiting the aptamer conformation-switching mechanism instilled through the selection process. First, we developed electrochemical aptamer-based sensors (E-ABs) to allow continuous FTC monitoring in vivo, as a tool for the study of FTC disposition in preclinical animal models.^[21]^ To create the E-ABs, we required FTC binding to induce conformation-switching in the aptamer. This is because, in E-ABs, target binding-induced conformation switching is used to modulate electron transfer between an aptamer-bound redox reporter and the electrode surface (Figure 4A), which is then measured via electrochemistry (typically square wave voltammetry). Because this sensing mechanism does not employ a complementary oligonucleotide like in the capture-SELEX strategy used to select the aptamer (Bio-Capture strand in Table S1, Supporting Information), it was critical to optimize the length of stem S1 in FTC_1_Full so that the aptamer remained unfolded in the absence of FTC, but the stem would be rapidly stabilized upon FTC binding.

Accordingly, we investigated the effect of serial truncations to stem S1 of FTC_1_Full (Figure 3A) on its FTC binding affinity, shortening it from 13 base pairs (bps) to 2 bps (Figure S5, Supporting Information). Remarkably, affinity for FTC was maintained across all truncations tested, indicating that a stem with as little as two bps (Figure 4A) was sufficient to allow proper folding of the aptamer and binding to FTC (Figure 4B). Similarly, aptamer selectivity was not affected by the truncations relative to cytosine, cytidine, and 5-fluorocytidine (Figure S6, Supporting Information). Additionally, a scrambled version of the aptamer showed no binding to FTC, further demonstrating high selectivity (Figure S7, Supporting Information).

To determine which aptamer truncation generated the largest effect in E-AB sensor signaling output (i.e., the largest conformational change), we synthesized all truncated variants with a 5′ alkylthiol linker and a 3′ methylene blue reporter modification as indicated in the Methods. Using these modified aptamers, we built E-AB sensors and interrogated them via square wave voltammetry to create frequency maps (Figure S8, Supporting Information), in the presence and absence of FTC. These measurements generate volcano plots with maxima that reflect the average rate of electron transfer of a given E-AB sensor. If FTC binding induces structure switching in the aptamer, the maxima should shift to higher square wave frequency values.^[22,23]^ Based on these measurements, we determined that the aptamer sequence with only two base pairs in the stem (Figure 4A) achieved the largest change in electron transfer upon FTC addition (Figure 4C vs Figure S8A–D, Supporting Information). This sequence had 19 nucleotide truncations from the 5′ terminus and 25 truncations from the 3′ terminus, expressed as FTC_1_5′ trunc19_3′trunc25 in Table S1 (Supporting Information), but hereby referred to as FTC_1_EAB for simplicity. By fabricating E-AB sensors with sequence FTC_1_EAB and challenging them with monotonically increasing concentrations of FTC in phosphate-buffered saline, we built dose-response curves (Figure 4D) that successfully covered the clinically relevant range of FTC concentrations, 100 nm to 10 μm, with a sensor gain at the high concentration of ≈200%. To confirm specificity for the E-AB platform, control dose-response curves were performed with FTC-triphosphate and lamivudine (Figure S9, Supporting Information) revealing negligible signal change across the 100 nm to 10 μm range. We did observe binding to lamivudine at concentrations higher than 10 μm, but with a net sensor gain 50% lower than that observed with FTC.

**Figure 5.**
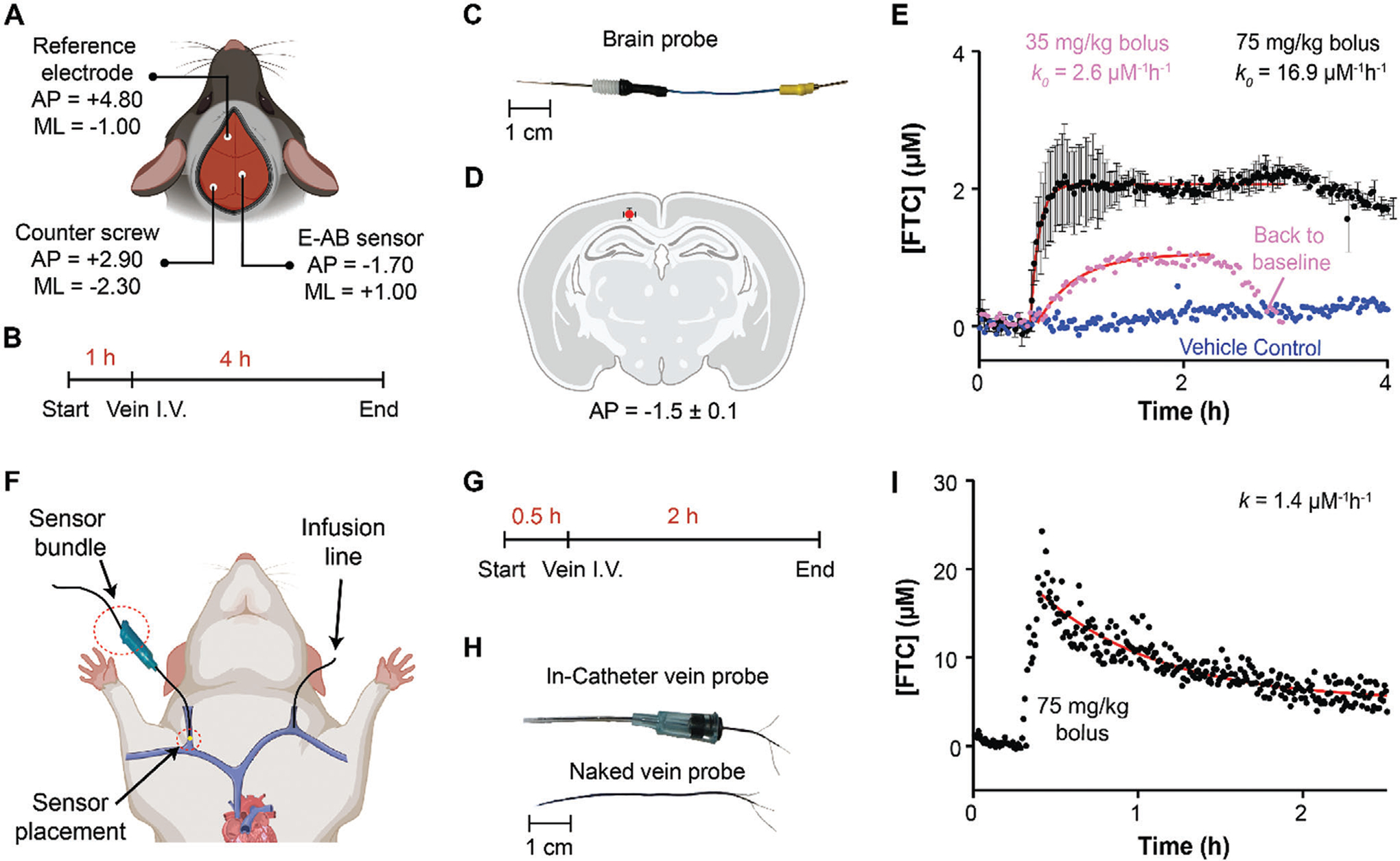
Pharmacokinetic measurements of FTC in preclinical animal models. A) Schematic of target sensor coordinates for mouse in-brain measurements. B) Protocol timeline. C) Photograph of brain E-AB probe. D) Confirmation of sensor placement in the cortex via histology. The red circle represents the average of 3 independent sensor placements, errors show the standard deviation. A representative brain scan post-implantation is shown in Figure S10 (Supporting Information). E) In-cortex E-AB measurements show an immediate increase in FTC concentration following intravenous dosing at either 75 mg kg^−1^ (*N* = 2, absorption rate, *k*_*0*_ = 16.9 μm^−1^ h^−1^) or 35 mg kg^−1^ (*N* = 1, absorption rate, *k*_*0*_ = 2.6 μm^−1^ h^−1^) as the drug crosses the blood-brain barrier. Solid black circles represent the average of two measurements; errors show the dispersion across such measurements. Solid pink circles represent a single measurement at the low dose. One surgery was performed per animal. F) Schematic of sensor insertion into rat jugular vein. G) Protocol timeline of in-vein measurements. H) Vein E-AB probe. I) In-vein E-AB measurements show an instantaneous increase in FTC, as expected given the intravenous bolus, followed by first-order excretion kinetics, *k* = 1.4 μm^−1^ h^−1^.

To demonstrate the ability of FTC_1_EAB to support E-AB sensing in vivo in continuous mode, we used this aptamer to fabricate brain probes as previously reported by our group.^[24]^ We wanted to demonstrate the E-AB measurement of FTC transport from blood to the brain across the blood-brain barrier because this drug and its metabolites may have implications in neurodegenerative processes in HIV patients undergoing FTC therapy. For this proof-of-concept demonstration, we placed the E-AB sensors in the cortex of mice (Figure 5A) and dosed FTC at 75 and 35 mg kg^−1^ via an intravenous bolus. Our measurement protocol (Figure 5B) consisted of placing the FTC E-AB sensor in the brain and starting the recording of a sensor baseline for 1 h, interrogating the sensor every 83 s. After this period, we dosed FTC via the tail vein and continued performing FTCmeasurements for a total of 4 h. An optical photograph of the E-AB brain probes is shown in Figure 5C. To confirm correct sensor placement in the brain, at the end of the measurements and prior to animal euthanasia we passed a current of 300 μA through the sensor for 2 s to locally burn the tissue, allowing us to determine sensor placement location via histology (Figure 5D and Figure S10, Supporting Information).

The resulting measurements (Figure 5E, black data, pink data) revealed a quick uptake of FTC into the brain cortex shortly (within minutes) after intravenous dosing of FTC. As a negative control, we dosed the rodents with the bolus vehicle, phosphate-buffered saline, without FTC, and observed a flat baseline with minimal drift (Figure 5E, blue data). The cortex FTC pharmacokinetic profile showed transport saturation with a plateau at ≈2.0 ± 0.1 μm following 75 mg kg^−1^ injection (*N* = 2), and a plateau at ≈1.1 μm following a 35 mg kg^−1^ bolus (*N* = 1). The red lines in Figure 5E illustrate non-linear regression of the data to a model of continuous infusion. These results indicate the shape of the profile is dose-dependent; specifically, the time to plateau concentrations and the duration of the steady state period changed with the dose delivered. Additionally, the results confirm the ability of our aptamer to support E-AB sensing in vivo in the brains of mice with high selectivity for FTC.

As an additional demonstration, we performed measurements in the vein of rats (Figure 5F) following previously published methods (Figure 5F, see Methods).^[25]^ The measurement protocol (Figure 5G) in this case was shorter because the overall drug pharmacokinetics were faster than those observed in the brain. We recorded a 30 min baseline followed by an intravenous FTC bolus, and continued monitoring for 1 h post-dosing.

For these measurements, we fabricated E-AB sensors in wire bundle format and encased them into 22G medical-grade catheters, as previously reported (Figure 5H).^[26]^ We then performed surgery on the rats to symmetrically dissect the external jugular veins. The right vein was used for sensor placement and the left vein for FTC dosing (Figure 5F). In contrast to the profiles measured in the brain, which plateaued at ≈2 μm and ≈1 μm following doses of 75 and 35 mg kg^−1^, respectively, the intravenous dose of 75 mg kg^−1^ achieved an order-of-magnitude higher concentration, C_MAX_ of ≈20 μm (*N* = 1), followed by rapid, first-order excretion kinetics, with a half-life of t_1/2_ ≈50 min (Figure 5I). These results reflect expected differences in drug levels between compartments because the blood-brain barrier should limit the absorption of FTC into the brain. The results also confirm different pharmacokinetics between compartments. Unfortunately, given the noise level observed in the baseline measurement in Figure 5E and in the vein data (Figure 5I), it is clear our E-AB sensor will not resolve human clinical levels at a steady state, i.e., 160 nm. However, the platform should still be useful for the study of drug-drug interactions. For example, it is known that cocaine use in patients undergoing FTC therapy can raise FTC uptake into the brain by 200%,^[21]^ a concentration change that should be easily detectable by our electrochemical platform. This application will be investigated further in future work. Also, future work beyond the scope of this study will seek out quantitative validation of our in vivo measurements relative to benchmark methods such as immunoassays.

We note that the discussed E-AB sensors are not without limitations. Because no proteins were included in the selection buffer during FTC aptamer enrichment, the FTC aptamer binds serum albumin. To illustrate this, we performed ITC measurements using bovine serum albumin (BSA) as the ligand; these measurements revealed a reproducible heat change between FTC_1 and BSA at mole ratios <0.1 (Figure S11A, Supporting Information). However, we overcame this limitation in our in-vein, in vivo measurements by encasing the E-AB probes in medical grade catheters filled with phosphate-buffered solution, which allowed the rapid exchange of FTC during our measurement period (Figure 5I) while filtering proteins via diffusion across the buffer blank, without significant sensor fouling by albumin. The in-brain measurements successfully employed bare sensors without buffer filtering, an indication that albumin levels in brain tissue are negligible. Alternative sequences from the enriched aptamer pool that showed less albumin binding (Figure S11B–D, Supporting Information), could not be successfully reengineered to undergo FTC-binding induced structure switching that was functional in the E-AB platform.

**Figure 6.**
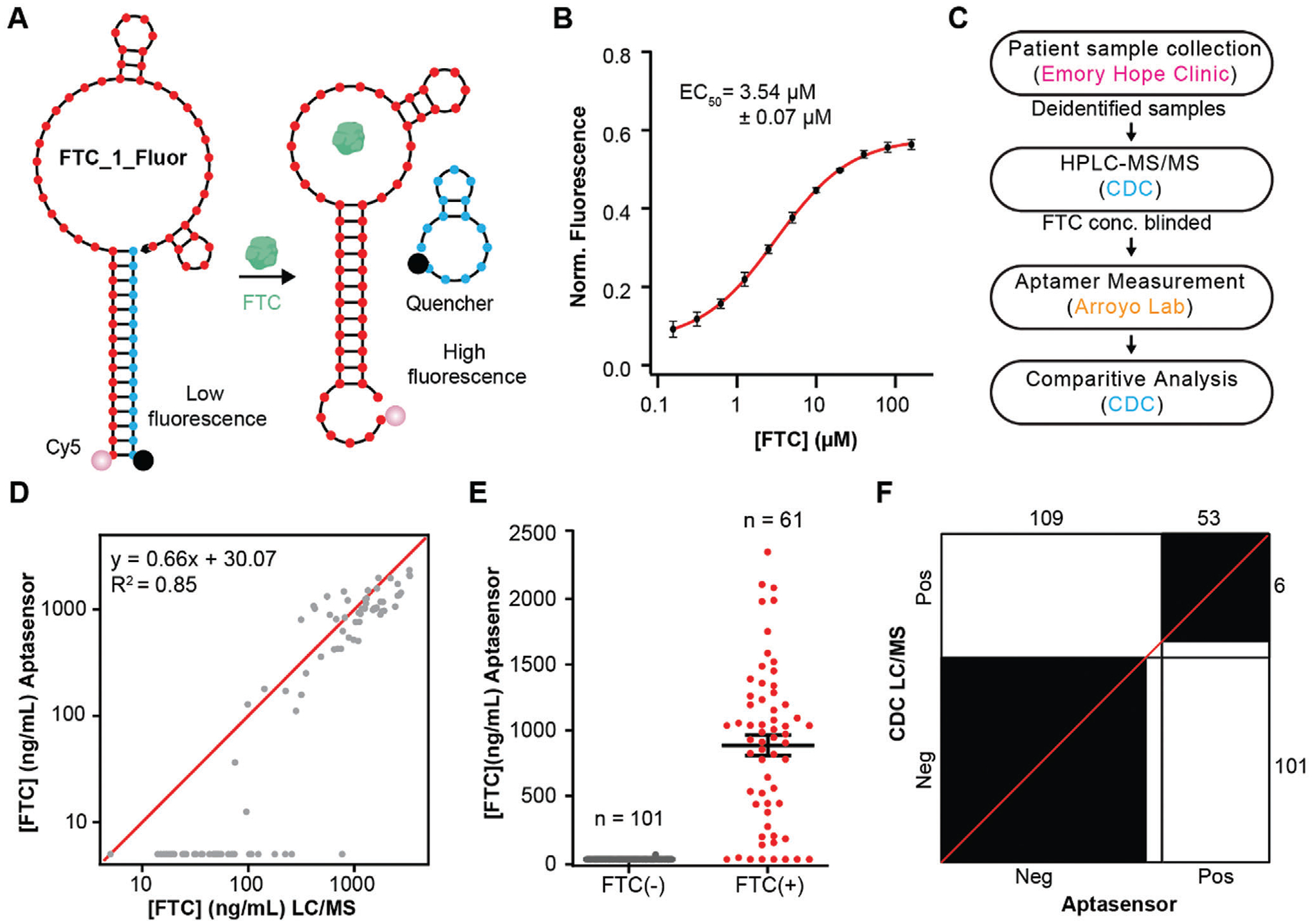
Fluorescence-based FTC Measurements in Human Plasma. A) Mode of operation of fluorescent FTC_1_Fluor sensor. B) Dose-response curve of FTC_1_Fluor exposed to FTC in 50% plasma. Solid circles show the average, errors the standard deviation of triplicate measurements. C) Blinded analytical validation protocol revealing sample collector, blinded LC-MS measurements at Centers for Disease Control (CDC), blinded measurements via FTC_1_Fluor, and the correlative analysis at CDC/Johns Hopkins. D) Differential scatter plot of FTC concentrations across *N* = 162 clinical samples determined via either FTC_1_Fluor (Y axis) or LC/MS (*X* axis). The red line represents a linear fit to “true positives,” which were considered samples with [FTC] > 96 ng mL^−1^, based on published mean trough plasma concentrations during daily dosing^[27]^ (E) Stratification of FTC_1_Fluor results relative to LC-MS confirmed positive/negative samples. The threshold for FTC positive is 96 ng mL^−1^. Zeros in positive cohort reveal patients undergoing ARV therapy that did not show measurable FTC levels at the current limit of detection. The horizontal line represents the mean ± standard errors for the two groups. F) Bangdiwala agreement chart of the FTC measurement comparing fluorescence assay measurements with mass spectrometry measurements. A 86.9% positive and a 100% negative percent agreement were observed. The threshold for FTC positive is set as 96 ng mL^−1^.

Like E-AB sensing, the reporting mechanism for our fluorescence-based sensor relied on FTC-induced structure switching (Figure 6A). In this case, the capture strand (Bio-Cap_1) originally employed during in vitro selection was re-purposed to quench fluorescence upon binding to the dye-labeled aptamer. When challenged with FTC, the formation of the aptamer-FTC complex was expected to promote displacement of the quencher-labeled capture strand (Q-Cap_1) and closure of the aptamer stem S1, leading to increased fluorescence emission. Additionally, we truncated 14 nucleotides from the 3′terminus of the FTC_1_Full aptamer to eliminate unpaired nucleotides extending from stem S1 upon FTC binding (see Figures 3A and 6A). These truncations also destabilized stem S2 in the unfolded aptamer which could impair structure switching (Figures S12 and S13, Supporting Information). The resulting aptamer-based fluorescent sensor is hereby referred to as FTC_1_Fluor (FTC_1_5′trunc3_3′trunc14 in Table S1, Supporting Information).

We showed that the affinity and selectivity of FTC_1_Fluor for FTC was comparable to the parent aptamer FTC_1_Full (Figures S14 and S15, Supporting Information). Importantly, FTC_1_Fluor was stable in 50% human plasma for up to 24 hours (Figure S16, Supporting Information) and the addition of up to 50% human plasma had little effect on sensor performance (Figure S17, Supporting Information), demonstrating the ability of FTC_1_Fluor to support FTC sensing in human biofluids. A kinetic analysis revealed that the maximum fluorescent signal was obtained within 1 min following exposure of FTC_1_Fluor to FTC in 50% human plasma, highlighting the rapid operation of this sensing platform (Figure S18, Supporting Information). Finally, we obtained a standard curve for FTC detection in 50% human plasma, revealing a limit of detection (LOD) of 0.046 ± 0.001 μm, a limit of quantification (LOQ) of 0.156 ± 0.005 μm, and a half-maximal effective concentration of binding (EC_50_) of 3.536 ± 0.069 μm (*R*^2^ = 0.99) (Figure 6B). Unlike Full_1_EAB, the FTC_1_Fluor sequence hybridized with Bio-Cap_1 showed no albumin binding. We confirmed this property via ITC (Figure S11E, Supporting Information).

To validate the precision of our high-throughput, FTC_1_Fluor for clinical use, we obtained 162 deidentified, FTC-concentration-blinded patient plasma samples collected from men who have sex with men (MSM) receiving FTC as part of clinical pharmacokinetic studies. We followed the validation study protocol shown in Figure 6C. First, the samples were deidentified and measured for FTC levels at the Centers for Disease Control and Prevention (CDC), using the benchmark standard LC-MS assay. Then, the samples were blinded and shipped to the Arroyo lab at the Johns Hopkins University School of Medicine. We screened these samples for FTC concentration using our optimized assay conditions (see Methods). For these measurements, we employed 384-well black plates. We added 20 μL plasma volume from each of the 162 specimens into individual wells. We then diluted with a 20 μL buffer solution of the FTC_1_Fluor/Q-Capture probe to a final concentration of 100 nM/150 nM (2-fold dilution factor). After 5 min, the plates were interrogated using a benchtop plate reader. Each plate contained an internal calibration curve, shown in Figure 6B for the data reported in this work. The resulting fluorescence emission intensities were normalized and converted to FTC concentration, as discussed in the Methods. The aptamer-measured concentrations were then mailed to the CDC labs for differential comparison against LC-MS measurements. The resulting lateral comparison is tabulated in Table S3 (Supporting Information). FTC concentrations measured by our fluorescence assay were in excellent agreement with benchmark LC-MS measurements (R^2^ of 0.85, Figure 6D, based on an empirical threshold for positives of 96 ng mL^−1^ as defined in the next paragraph). Additionally, our fluorescent assay achieved 100% correlation in reporting true negatives (i.e., it reported no false positives, Figure 6E).

To stratify the patient sample into FTC positive versus FTC negative groups, we empirically set an FTC threshold at 96 ng mL^−1^, based on published mean trough plasma concentrations during daily dosing.^[27]^ This exercise resulted in positive percent (PPA) and negative percent agreements (NPA) of 86.9% and 100%, respectively, relative to LC/MS measurements (Figure 6F). The overall rate of agreement (ORA) was 95%. To further demonstrate the specificity of our fluorescence assay, we built a receiver operating characteristic curve (ROC curve), which gave a 94.2% area under the curve (AUC) score (Figure S19, Supporting Information). This ROC analysis also confirmed that the threshold for a positive sample was set properly for our assay. Taken together, the data demonstrate that our fluorescent assay achieves similar performance relative to benchmark LC-MS methods, but in a fraction of the time and in a high throughput format.

## 3. Ethical Approvals

Peripheral blood was collected from HIV-negative male participants in two clinical studies registered at clinicaltrials.gov and conducted at the Emory Hope Clinic in Atlanta, Georgia.^[28,29]^ Both trials were funded by the US Centers for Disease Control and Prevention (CDC) and approved by Emory University and CDC Institutional Review Boards. All study participants gave written informed consent, and the trials conform to the US Federal Policy for the Protection of Human Subjects.

All animal procedures were approved by the Johns Hopkins University Animal Care and Use Committee (Protocols: MO22M234, RA22M242) and in accordance with the Guide for the Care and Use of Laboratory Animals.

## 4. Conclusion

This study shows a complete translational workflow of two aptamer-based sensors. We started by identifying a valuable clinical application, in this case, monitoring adherence to ARV therapy. Then, we carried out aptamer development, biophysical characterizations, and translation to two biosensing platforms: one for in vivo molecular monitoring, and a second one for single-point ARV concentration measurements. The results of this work highlight the current maturity of aptamer technology and the high value it can bring to clinical practice which, by currently being limited to analysis via costly, benchtop instrumentation at centralized facilities, lacks the flexibility to support population-level screening of ARV.

There are limitations to our study. First, only a training set of 162 samples was used to benchmark the performance of our fluorescent aptamer biosensor in determining FTC concentrations relative to LC-MS. A study with a larger cohort of specimens should be completed to further confirm the analytical accuracy and clinical value of our biosensor platform. Additionally, this study only looked at FTC concentrations; however, the platform could be expanded to the monitoring of 3TC (lamivudine), which is commonly used in place of FTC in ARV regimens. For in vivo research applications, the platform could also be expanded to support the independent monitoring of FTC triphosphate, the main metabolite of FTC, which could offer unprecedented insights into FTC metabolism in tissues and drug-drug interactions.

Given the advanced stage of the reported FTC sensors, future efforts from our laboratories will focus on continuing clinical translation via formal pilot trials with larger patient cohorts. We will also seek to evaluate the applicability of our FTC_1_Fluor to other sample matrices collected for assessing adherence, including urine, and blood spots.

## Supplementary Material

Supplementary Info

## Data Availability

The data that support the findings of this study are available from the corresponding author upon reasonable request. The data can also be accessed at the following DOI: http://doi.org/10.7281/T11IAN9N.
