# Supplementary material for: Monitoring HIV Antiretroviral Therapy via Aptamer-Based Measurements in Preclinical Animal Models, in Human Plasma
Source: Adv Sens Res. Author manuscript; Available in PMC 2025 Sep 30. (PMC12478587; doi:10.1002/adsr.202400191)
Supplement: Supplementary Info [file NIHMS2064577-supplement-Supplementary_Info.docx]

**SUPPLEMENTARY INFORMATION**

**Monitoring HIV Antiretroviral Therapy via Aptamer-Based Measurements in Preclinical Animal Models and in Human Plasma**

Jing Li,^1,†^ Vincent Clark,^2,†^ Chen-Hsu Yu,^1^ Karen Scida,^3^ Miguel Aller Pellitero,^4^ Rolando L. Albarracín Rivera,^1^ Wenrui Zhong,^1^ Erin Demek,^2^ Jeffrey Fountain,^5^ J.D. Mahlum,^2^ Richard E. Haaland,^5^ Gregory V. Carr,^3,4^ Jonathan Sczepanski,^1,*^ and Netzahualcóyotl Arroyo-Currás^2,4,*^

^1^ Department of Chemistry, Texas A&M University, College Station, TX 77842, United States

^2^ Chemistry-Biology Interface Program, Zanvyl Krieger School of Arts & Sciences, Johns Hopkins University, Baltimore, MD 21218, United States

^3^ Lieber Institute for Brain Development, Johns Hopkins University School of Medicine, Baltimore, MD 21205, United States

^4^ Department of Pharmacology and Molecular Sciences, Johns Hopkins University School of Medicine, Baltimore, MD 21205, United States

^5^ Division of HIV/AIDS Prevention, Centers for Disease Control and Prevention, Atlanta, GA 30329, United States

^†^ Authors contributed equally to this work.

***Correspondence to:**

Netz Arroyo, Ph.D.

Hunterian Building, Room 316

Johns Hopkins University School of Medicine

725 North Wolfe St.

Baltimore, MD 21205

netzarroyo@jhmi.edu

(443) 287-4798

Jonathan T. Sczepanski, Ph.D.

Chemistry Building, 3255 TAMU

Texas A&M University

580 Ross St

College Station, TX 77843

jon.sczepanski@chem.tamu.edu

(979) 862-3731

Table of Contents

[Materials and Methods 3](#_Toc183118294)

[Table S1: DNA sequences used in this study. 14](#_Toc183118295)

[Table S2: Detailed conditions used for FTC aptamer in vitro selection. 15](#_Toc183118296)

[Table S3: Lateral comparison of measured sample concentrations. 16](#_Toc183118297)

[Fig S1: Monitoring capture-SELEX progress by elution profiles 22](#_Toc183118298)

[Fig S2: Clone SELEX elution profiles of 10 candidates. 23](#_Toc183118299)

[Fig S3: Sequencing results of round 11 pool from aptamer selection. 24](#_Toc183118300)

[Fig S4: Binding of FTC to top cluster sequences via ITC. 25](#_Toc183118301)

[Fig S5: Serial truncations of FTC_1_Full maintain FTC binding. 26](#_Toc183118302)

[Fig S6: ITC thermograms of FTC_1_EAB against close analogs of FTC. 27](#_Toc183118303)

[Fig S7: A scrambled version of FTC_1_EAB displays no binding to FTC 28](#_Toc183118304)

[Fig S8: Frequency maps of FTC_1 truncations. 29](#_Toc183118305)

[Fig S9: Dose response curves of FTC_1_EAB against structural analogs. 30](#_Toc183118306)

[Fig S10: Representative histology image from in vivo brain experiments. 31](#_Toc183118307)

[Fig S11: ITC thermograms of FTC binding aptamers against BSA 32](#_Toc183118308)

[Fig S12: Mechanism of action of FTC_1_Fluor. 33](#_Toc183118309)

[Fig S13: Normalized fluorescence of modified constructs. 34](#_Toc183118310)

[Fig S14: ITC Thermogram of FTC_1_Fluor against FTC. 35](#_Toc183118311)

[Fig S15: Specificity of FTC_1_Fluor against dC. 36](#_Toc183118312)

[Fig S16: Stability in human plasma. 37](#_Toc183118313)

[Fig S17: Effect of human plasma on sensor performance. 38](#_Toc183118314)

[Fig S18: Kinetic analysis of measurement in 50% human plasma. 39](#_Toc183118315)

[Fig S19: Receiver Operating Characteristic curve (ROC curve) of the clinical assay. 40](#_Toc183118316)

[Fig S20: ESI-MS spectra of FTC_1_Fluor components. 41](#_Toc183118317)

[Fig S21: ESI-MS spectra of FTC_1_EAB + Surface Modifiers. 42](#_Toc183118318)

# Materials and Methods

**Materials.** Oligonucleotides were either purchased from Integrated DNA Technologies (Coralville, IA) or prepared by solid-phase synthesis on an Expedite 8909 DNA/RNA Synthesizer. Solid-phase oligonucleotide synthesis reagents, including nucleoside phosphoramidites, 3′-PT-Amino-Modifier C6 CPG, thiol-modifier C6 S−S phosphoramidite, MB NHS ester, and Glen-Pak DNA purification cartridges, were purchased from Glen Research (Sterling, Va). All oligonucleotides were purified by denatured polyacrylamide gel electrophoresis (PAGE), desalted using an Amicon Ultra-15 Centrifugal Filtration Unit (3kDa MWCO), and ethanol-precipitated prior to use. Taq DNA polymerase was expressed and purified according to literature procedures *(25)*. MyOne^TM^ Streptavidin C1 magnetic Dynabeads, high-capacity streptavidin agarose beads (50% slurry), and SuperScript II Reverse Transcriptase were purchased from Thermo Fischer Scientific (Waltham, MA). Deoxynucleotide triphosphates (dNTPs), deoxycytidine, FTC (PHR2120-500MG), and 24 mm x 60 mm cover glasses were purchased from Sigma Aldrich. Granular paraformaldehyde, 0.1% w/v Cresyl violet, Permount mounting medium, and Xylene substitute Hemo De purchased from Electron Microscopy Sciences (Hatfield, PA). Superfrost Plus^TM^ glass slides, sodium azide (NaN_3_), sucrose, and 100% reagent grate alcohol were obtained from VWR (Radnor, PA). All the other chemicals were purchased from either Sigma-Aldrich, Alfa Aesar or Fisher Scientific and used as received. Phosphate buffered saline (PBS) (46-013-CM) was purchased from Corning Inc (Corning, NY). All other buffers were prepared in house using Milli-Q purified water (18.2 MΩ) and filtered using a 0.22 μm membrane prior to use.

**Capture SELEX.** 200 picomoles of a single-stranded DNA library (all sequences used during capture SELEX are listed in Table S1) was amplified by PCR in a 10 mL reaction volume containing 0.5 μM of each primer (Fwd_1 and Rev_1), 50 mM KCl, 1.5 mM MgCl_2_, 0.1% TRITON-X, 10 mM Tris (pH 9.0), 0.5 mM each of the four dNTPs, and 0.05 U/μL Taq DNA Polymerases. The PCR reaction was carried out using the following temperature cycling: i) 95° C for 3 min, (ii) 95° C for 30 s, (iii) 59° C for 30 s, (iv) 72° C for 1 min, and (v) 72° C for 5 min, with repeating steps (ii) to (iv) for 5 total cycles. The resulting dsDNA products were precipitated by ethanol, redispersed in 100 μL TE buffer (10 mM Tris (pH 7.6), 1 mM EDTA), and then added directly to 500 μL of settled high-capacity streptavidin coated agarose beads in wash buffer (WB; 100 mM NaCl, 50 mM Tris, pH 7.6). After incubating at room temperature for 30 min, the beads were washed twice with 500 μL WB buffer and once with 500 μL water. The nonbiotinylated strands were then eluted from the beads using 2 × 175 μL of ice-cold elution buffer (EB, 50 mM NaOH, 1 mM EDTA). The eluent was immediately neutralized by the addition of 35 μL 1 M Tris (pH 7.6) and 35 μL 3 M NaOAc, and the resulting mixture was precipitated by ethanol. The obtained ssDNA library was further purified by denaturing PAGE (10%, 19:1 acrylamide:bis-acrylamide) prior to use.

Meanwhile, 200 μL streptavidin coated magnetic beads (slurry) were washed following manufacturer’s instructions and the non-specific sited were blocked using 500 μL of SELEX buffer (SB; 1x PBS, 2 mM MgCl_2_) containing 1 mg/mL yeast tRNA. The ssDNA library prepared above (Lib_1) and a capture oligonucleotide (Bio-Cap_1) were mixed at a 1:5 molar ratio in 250 μL of SB buffer. The DNAs were heated to 98^o^ C for 5 min and allowed to slowly cool to room temperature. The library/capture oligonucleotide mixture was then added to the prepared magnetic beads and the slurry was allowed to incubate at room temperature for 30 minutes with gentle shaking. The beads were then washed 10 times with 1 mL SB buffer to remove unbound or weakly bound sequences. The length of each washing step is listed in Table S2 and varied per round. After washing, functional aptamers were eluted from the beads using 3 × 250 μL washes with SB buffer containing FTC (this is referred to as the positive selection step). Again, the length of each washing step and the FTC concentration used is listed in Table S2 and varied per round. The eluent from each of the three positive selection washes were combined, precipitated by ethanol, and the resulting pellet was dissolved in 100 μL water. Half of this DNA was used as template for PCR to generate the enriched ssDNA library for the next selection round (as described above). For rounds 9 and onward, a negative selection step was introduced following the initial 10 × 1 mL SB buffer washes. Here, non-selective aptamers were eluted from the beads using 3 × 1 mL washes with SB buffer containing 50 μM deoxycytidine (see Table S2). The washed beads were then progressed directly into the positive selection step. To track the selection process, a small-scale PCR was run during each selection round to generate an elution profile using the eluant from (1) the first and last washes from the initial 10 × 1 mL SB washing step, (2) the final wash of the negative selection step (if applicable), and (3) all three washes from the positive selection step. Representative data is shown in Fig. S1.

**DNA Sequencing*.*** The ssDNA eluted from SELEX round 11 was amplified as described above and purified in a 2% agarose gel. The purified DNA was subjected to a second PCR round using sequencing primers (Table S1). Next-generation sequencing was carried out at the Johns Hopkins School of Medicine Genetic Resources Core Facility using an Illumina MiSeq System. Sequence reads were analyzed and clustered using the FASTAptamer toolkit *(15)* (Levenshtein edit distance of 4 nucleotides).

**Isothermal Titration Calorimetry (ITC).** All measurements were performed using an Affinity ITC (TA Instruments, New Castle, DE) coupled to an autosampler and associated software. All target and aptamer solutions were first prepared in PBS + 2 mM MgCl_2_ followed by 10 min of degassing prior to ITC measurements. Before each titration, the system was cleaned three times with deionized water, followed by a 300 s equilibration in the same buffer. The cell was loaded with 20 µM of the respective aptamer, and the syringe was loaded with 200 µM of the appropriate target. Heat-exchange was recorded after a 1 µL injection of target, over 35 injection points, with 150 – 180 s of equilibration time between each injection. All titrations were carried out at 25° C. Experimental and cleaning protocols may be found in the data repository associated with this publication.

**Synthesis of Fluorescent Sensor FTC_1_Fluor.** The truncated aptamer FTC_1_Fluor was purchased from Integrated DNA Technologies (Coralville, IA) with a 5′ amino modifier (5' Amino Modifier C6). The quencher strand Q_Cap_1 was prepared by solid-phase synthesis on an Expedite 8909 DNA/RNA Synthesizer using a 3′-Black Hole Quencher 2 (BHQ2)-CPG according to manufacturer protocol. Sulfo-Cy5 N-hydroxysuccinimide (NHS) ester (Lumiprobe Corp., Hunt Valley, MD) was conjugated to the 5ʹ end of FTC_1_Fluor. Conjugation reactions were performed by combining the 5′ amino modified oligonucleotide (100 μM) with the dye NHS ester (5 mM final concentration) in 0.1 M sodium bicarbonate buffer (pH 8.5). The reaction was vortexed intermittently over a 16-hour period at 23°C. Samples were then ethanol precipitated and the labeled oligonucleotide was purified by 20% denaturing PAGE (19:1 acrylamide:bisacrylamide). Purified oligonucleotides were excised from the gel and eluted overnight at 23 °C in elution buffer (200 mM NaCl, 10 mM EDTA, and 10 mM Tris (pH 7.6)). The solution was then filtered to remove gel fragments, and eluted oligonucleotides were desalted by ethanol precipitation and an Amicon Ultra Centrifugal Filter 3KDa (MilliporeSigma, Burlington, MA). The concentration was determined by absorbance at 260 nm on a NanoDrop 2000c (ThermoFisher, Waltham, MA). The identity of the modified aptamer was confirmed by mass spectrometry (Fig. S20).

**Fluorescent Biosensor Measurements**. We first prepared a 4X assay master mix and a 4X control master mix containing all required components. The 4X assay master mix was composed of 400 nM Cy5-aptamer FTC_1_Fluor and 600 nM Q_Cap_1 in buffer F (4x PBS and 4 mM MgCl_2_). The 4X control master mix was identical but lacked Q_Cap_1. Prior to use, both the assay mix and control mix were heated to 95°C for 5 minutes, and slowly cooled to 22º C to anneal the aptamer and capture-strand components. A standard curve was generated by conducting the fluorescence assay using a two-fold dilution of FTC in human plasma. In brief, a solution of 320 µM FTC in plasma was first prepared, followed by two-fold serial dilution with plasma to generate a set of standard samples for calibration. 10 µL of either the 4X assay or control master mix was diluted with 10 µL H_2_O and transferred to a black 384-well plate (Corning #3575). 20 µL of the indicated FTC standard plasma sample was added to the assay mixtures. Plasma without FTC was added to the control assay mixtures. After 5 min, the fluorescence was measured on a GloMax Discover multi-well plate reader (Ex: 627 nm; Em: 660-720 nm) or a Molecular Devices SpectraMax ID5 (Ex: 627 nm; Em: 670 nm). All fluorescence was normalized to the control assay.

The standard curve was generated by curve fitting in GraphPad Prism (v.9.3.1) using one-phase exponential model, according to eq. 1*(30)*:

$\left[ FTC \right]= \frac{-1}{K} \ln\frac{Y-Y_{Max}}{Y_{Blank}-Y_{Max}}$ eq. 1

where Y represents the normalized fluorescence of measurement, Y_Max_ represents the maximum normalized fluorescence, Y_Blank_ represents the normalized background fluorescence, K represents the concentration scaling factor. Limit of detection (LOD) and limit of quantification (LOQ) were determined by performing fluorescence assays with 15 blank plasma samples. LOD was defined as the average fluorescence of blank plus 3 standard deviations of the blank and LOQ was determined as the average fluorescence of blank plus 10 standard deviations of the blank measurement. The calculated fluorescence value for LOD and LOQ was converted into concentration using the above-described standard curve. Kinetic assays (Fig. S16) were carried out similarly and were monitored for 40 minutes at 22º C.

**Plasma Stability Assay.** A 40 µL reaction mixture was prepared as described above containing 50% human plasma with or without 10 µM FTC. At the indicated times, a 3 µL aliquot was taken and quenched with 12 µL denaturing PAGE loading buffer (90% formamide, 10 mM EDTA). The quenched solution was then resolved by 20% denaturing PAGE (19:1 acrylamide:bisacrylamide) and imaged by a Typhoon FLA9500 Multimode Imager (Cy5; excitation/emission: 649 nm/670 nm).

**Clinical Samples.** Peripheral blood was collected from HIV-negative male participants in two clinical studies registered at clinicaltrials.gov and conducted at the Emory Hope Clinic in Atlanta, Georgia. (*Main text references 28, 29*) Both trials were funded by the US Centers for Disease Control and Prevention (CDC) and approved by Emory University and CDC Institutional Review Boards. All study participants gave written informed consent, and the trials conform to the US Federal Policy for the Protection of Human Subjects. In one study, 40 male participants provided 108 blood specimens after receiving a single oral dose formulation containing 200 mg emtricitabine (FTC), 10 mg tenofovir alafenamide (TAF), 150 mg elvitegravir (EVG) and 150 mg cobicistat (COBI) as well as a single 800 mg dose of darunavir (DRV) (NCT03472963). In the second study, 31 male participants provided 54 blood specimens after receiving two doses of a single formulation containing 200 mg FTC, 10 mg TAF, 150 mg EVG and 150 mg COBI given 24 hours apart (NCT03976752). Peripheral blood specimens were collected in sodium citrate cell preparation tubes (CPT) (Becton Dickinson, Franklin Lakes, New Jersey, USA). Plasma aliquots were collected from CPT following centrifugation. Concentrations of FTC were measured in plasma specimens using high-performance liquid chromatography-tandem mass spectrometry (HPLC-MS/MS) (Sciex, Foster City, CA, Shimadzu Scientific Instruments, Durham, NC) as previously described. *(31, 32)*

**Aptamers for Electrochemical Sensors.** Aptamers for electrochemical sensing were functionalized with a 6-carbon disulfide group (thiol-modifier C6 S-S, Glen Research) at their 5′ terminus and two methylene blue (MB) units at their 3′ terminus. Solid-phase synthesis was initiated using 3´-PT-Amino-Modifier C3 CPG (Glen Research) followed by coupling of a TFA-Amino C7 Multiaddition CE-phosphoramidite (BioSearch Technologies), allowing for the eventual conjunction of two MB molecules to the 3′ terminus. Following synthesis of the aptamer sequence (Table S1), a 6-carbon disulfide group (thiol-modifier C6 S-S, Glen Research) was added to the 5′ terminus. The terminal 4,4′-dimethoxytrityl (DMT) group was retained for downstream purification. The synthesized aptamers were deprotected using AMA (ammonium hydroxide/40% aqueous methylamine, 1:1 v/v) and concentrated using a Glen-Pak DNA purification cartridge following the manufactures instructions. The deprotected aptamers were then purified by 20% denaturing PAGE (19:1 acrylamide/bisacrylamide). Purified aptamers were excised from the gel and eluted overnight at 23 °C in a buffer consisting of 200 mM NaCl, 10 mM EDTA, and 10 mM Tris (pH 7.6). The suspension was then filtered to remove gel fragments, and eluted aptamers were concentrated using an a 3K Amicon® filter device (Sigma Aldrich, St. Louis, MO) and desalted by ethanol precipitation.

MB conjugation reactions were performed by combining the amino modified aptamer (100 µM) with the MB NHS ester (2 mM final concentration) in 20 µL of 0.1 M bicarbonate buffer (pH 9) containing 10% DMSO. The reaction was vortexed intermittently over a 16-hour period at 23 °C. The reaction mixture was then passed through a NAP-5 Sephadex G-25 Column (GE Healthcare, Chicago, IL) to remove excess MB and the modified aptamer was further purified by 20% denaturing PAGE as before. The identity of all MB-modified aptamers was confirmed by mass spectrometry (Fig. S21).

**Fabrication of In-Vivo Electrochemical Probes.** We fabricated two sensor form factors, one for deployment in the jugular vein of rats (i.e., for blood drug measurements) and one for in-brain measurements. *For blood measurements*, E-ABs were fabricated as described in previous reports*(22, 29)*. Segments of pure gold (7.75 cm in length), platinum (7.25 cm), and silver (6.75 cm) wire, were cut to make the sensor body. The insulation at both ends of these wires, about 1 cm, was removed using a surgical blade to allow electrical contact. These were then soldered each to one of the three ends of a connector cable using 60% tin/40% lead rosin-core solder (0.8 mm diameter) and then insulated by applying heat to shrinkable tubing around the body of the wires, except for a small window of about 5 mm at the edge of each wire. The electrodes bundle, where each is shorter than the other, was assembled by staggering the electrodes to prevent electrical shorts. To do this, first all three electrodes were aligned at their non-exposed end, second the gold wire was attached to the platinum electrode using shrink tubing, and, finally, the gold-platinum set was attached to the silver electrode via the same method. The sensor window (i.e., the region devoid of insulation) in the gold wire was cut to approximately 3 mm in length. *For brain measurements*, we fabricated probes by adapting the carbon-fiber electrode fabrication protocol from the field of fast-scan cyclic voltammetry to create gold-based microprobes for in-brain measurements *(30)*. Briefly, Au wire (50 µm in diameter, GoodFellow, Huntington, Eng) was inserted into a 75 µm ID/150 µm OD fused silica capillary (Molex Incorporated, Lisle, IL) and sealed on one end using two-part 3-5 min curing Double/Bubble epoxy (McMaster-Carr, Elmhurst, IL), with ~ 0.5 cm of Au wire protruding from each end of the capillary. This capillary/Au wire was then inserted inside a guide cannula (18GA cut 1mm below and 10 mm above pedestal, PlasticsOne Technologies, Roanoke, VA) and glued on both ends using the same epoxy, leaving 1 cm of the capillary/Au wire exposed on the long end of the guide cannula (side to be functionalized with the FTC aptamer) and ~300 µm on the other end. Electrical connection to the latter was achieved by soldering to a 30 AWG Insulated Kynar Copper Wire Roll cable (Amazon) which had been previously pealed on both ends to expose the conductive material (named hereafter as Cu-cable). The soldering process was aided with 280 µM in diameter shrink tubing (Zeus, Orangeburg, SC) to hold the capillary/Au wire protruding from the short end to the Cu-wire. The Cu-wire’s end opposite to the Au wire-connection was previously soldered to a Au pin. Finally, 28-26 AWG shrink tube (FP-301 thin wall, 3M, Saint Paul, MN) was used to protect and reinforce all soldered areas. The active sensor windows of the probes were approximately 500 μm-1 mm long and 50 µm in diameter. The reference electrodes were fabricated by soldering one extreme end of a Cu-cable to a Au pin and the other extreme end to a Ag wire. We added adhesive to the soldered area to provide more support and prevent accidental brain exposure to the solder material. Before implantation in the brain, we immersed ∼2 mm of the Ag wire in a solution of 0.3 M FeCl3 in 0.1 M HCl for 30 s to produce the Ag/AgCl reference electrode. For the counter electrodes, we soldered a stainless-steel screw to a Cu-cable.

**Rodent Surgical Procedures.** In-blood FTC measurements were performed in male Sprague-Dawley rats purchased from Charles River Laboratories (Code: 400, Severn, MD), weighing between 300 and 350 g. All animals were pair-housed in a standard light cycle room (08:00 on, 20:00 off) and allowed ad libitum access to food and water. The rats were induced under 4% isoflurane anesthesia in a Plexiglas anesthesia chamber. The rats were then maintained at 2−3% isoflurane gas for the duration of the experiment. While anesthetized, surgery was performed to emplace the E- AB sensor within the jugular veins. Briefly, the area above the left jugular vein was shaved and cleaned with betadine and 70% ethanol. A small incision was made above the vein, then the vein was isolated. A small hole was cut into the vein with spring-loaded micro scissors. We then inserted the E-ABs and tied them into place sterile silk suture (Fine Science Tools, Foster City, CA). Once E-AB measurements were concluded, the rats were overdosed on 5% isoflurane gas until breathing and heartbeat ceased and then decapitated using a guillotine. For in-brain measurements, we used male C57BL/6J mice (Strain #: 000664; The Jackson Laboratory, Bar Harbor, ME). Before surgery, we removed the mice from their home cage and placed them in an anesthesia box where we flowed O_2_ at 1 L/min and 5% isoflurane until the mouse was under surgical place of anesthesia (confirmed by lack of toe-pinch reaction). We shaved the hair off the head from eyes- to ears-level and, once we immobilized the head in the stereotaxic frame (Stoelting, Wood Dale, IL), we lowered the isoflurane level to 3% (always checking for lack of toe-pinch reaction). We applied lube to the eyes and betadine to the shaved area using a cotton-tip applicator before making an incision using a scalpel. We removed the connective tissue from the exposed skull using a cotton-tip applicator and added 3% hydrogen peroxide solution to aid in the visualization of the sagittal, bregma, and lambda suture lines (for alignment of the head with the stereotaxic frame). Next, we drilled three holes in the skull with the following coordinates:

1. Reference electrodes: +4.80 AP (from bregma), −1.00 ML (from bregma), and −2.00 DV (from brain surface)

2. E-ABs electrodes: −1.70 AP (from bregma), +0.50 ML (from bregma), and −1.00 DV (from brain surface)

3. Counter-electrode screws: −2.90 AP (from bregma), −2.30 ML (from bregma), and 3–4 full rotations inside the hole.

To find the brain surface with the E-ABs, we connected the Gamry Reference 600+ potentiostat to all three electrodes, lowered the E-ABs near the skull and centered them to their corresponding hole (coordinates #2 above). We initiated 10 consecutive cyclic voltammograms from 0 to 0.5 V at 0.01 V/s and slowly lowered the sensor tip toward the brain. When the sensor does not touch the brain, the cyclic voltammogram shows just noise because the electrical circuit is open. Once the tip of the sensor touches the brain, a spike in the cyclic voltammogram current is observed, indicating the circuit is closed. At this point, we zeroed the DV coordinate of the digital stereotaxic arm reader and lowered the electrode to its final depth inside the brain. Finally, we ran another “pretreatment” session of 60 square wave voltammograms from 0 to 0.5 V at a frequency of 300 Hz (2 mV step, 50 mV amplitude) to ensure the sensor is homogenized with the brain tissue before starting the experiment. All procedures were approved by the Johns Hopkins University Animal Care and Use Committee (Protocols: MO22M234, RA22M242) and in accordance with the Guide for the Care and Use of Laboratory Animals.

**Preparation of Electrochemical, Aptamer-Based Sensors (E-ABs).** Regardless of form factor, all electrochemical probes were prepared as follows: we electrochemically cleaned the in vivo probes using a Gamry Reference 600+ potentiostat (Warminster, PA) by performing 200 cycles using cyclic voltammetry first from −0.3 to −1.6 V (vs Ag|AgCl) in 0.5 M NaOH at a scan rate of 0.5 V/s, and then from 0 to 1.55 V (vs Ag|AgCl) in 0.5 M H2SO4 also at a scan rate of 0.5 V/s. We then increased the electroactive surface area of the electrodes by roughening their surface via chronoamperometry*(29)*. For this, we performed 300 pulses (0.01 s pulse width and lowest sensitivity) from 0 to 2 V (vs Ag|AgCl). This was repeated 100 times using a macro (for a total of 30,000 pulses). Finally, we performed an additional 20 cycles in H_2_SO_4_ (same conditions as during electrochemical cleaning) to ensure the stability of the roughened surface. While the electrodes were being cleaned and roughened, a 1 μL aliquot of 100 μM FTC aptamer solution was reduced with 2 μL of 5 mM TCEP for 1 h and then diluted to 500 nM using PBS. The probes were incubated in the diluted aptamer solution, immediately after electrochemical roughening, for 2 h at 22º C (manually stirred the electrode in the solution to homogenize the solution/electrode interface). Finally, the electrodes were removed from the aptamer solution and immediately transferred into 1 mM MCH at room temperature overnight. During both incubation periods, we protected the solutions from evaporation by covering the electrodes/solution with a petri dish that was taped to the bench to minimize air flow.

The next day, we removed the electrodes from the MCH solution and thoroughly rinsed with deionized water before placing them in a beaker with PBS solution. We then checked the quality of the sensors by performing square wave voltammograms from 0 to 0.5 V (vs Ag|AgCl) at 30 and 300 Hz (50 mV amplitude and 2 mV step size). At this point, we ran 100 consecutive square wave voltammograms at 300 Hz to “pretreat” the sensor and remove any nonspecifically bound aptamer from the electrode surface. We kept the sensors in this PBS solution until ready to implant in the rodents.

**In vivo FTC measurements.** For all in vivo measurements a 60 min sensor baseline was established before drug infusions. After establishing a stable baseline, FTC was infused at a dose of 75 mg/kg via tail-vein intravenous administration. E-AB recordings continued for up to 6 h before experiment termination and euthanasia. In-brain measurements were performed at 10 and 550 Hz, 50 mV amplitude and 2 mV potential step. At the end of each in-brain measurements, the mice were euthanized, and their brains were collected into 4% paraformaldehyde (PFA) and stored at 2-8 ºC until used. The real-time plotting and analysis of voltammetric data were carried out via SACMES, an open access script previously reported by our group *(33)*.

**Histology**. Brains were removed from 4% PFA and immediately immersed into 30% sucrose, containing 0.05% NaN_3,_ for 48 h. The brain tissue was then sliced (50 µm-thick slices) using a SM2010R Leica microtome (Wetzlar, Germany) and slices of interest were stored in 1x PBS for staining with Cresyl violet. To do this, the tissue was mounted on charged glass slides and allowed to dry overnight. Then, the glass slides were dipped in the following solutions: (1) 15 min in 95% ethanol, (2) 2 min in 70% ethanol, (3) 2 min in 50% ethanol, (4 and 5) 2x 3 min in distilled water, (6) 5-10 min in 0.1% Cresyl violet, (7) 2 min in distilled water, (8) 2 min in 50% ethanol, (9) short dips in 95% ethanol containing 1 mL of 10% acetic acid until stain clears to desired level, (10) 2x 3 min in 100% ethanol, and (11) 5 min in Xylene substitution Hemo De. One by one, a cover glass was then added with Permount mounting medium immediately after removing the glass slides from the Xylene substitution solution and allowed to dry overnight before sealing the edges with clear nail polish (Amazon). Finally, the stained tissue was analyzed with an Aperio CS2 Leica microscope scanner (Wetzlar, Germany) to find the lesions indicating E-AB sensor location during the in vivo measurements.

**Statistical Analyses**. All analyses were conducted as described in the individual sections above. However, a brief description is also included in the next subtopics: (1) Pre-processing of data: all data was pre-analyzed in excel or using SACMES, as indicated above. (2) Data presentation: the data in all cases is presented either as single measurements (for most ITC experiments) or as the mean of two to five replicates, as indicated in figure captions. Errors represent the spread in the data as standard deviations. (3) Sample sizes are noted in all figure captions. (4) Clinical data was analyzed and presented in four forms: as a scatter plot, to directly compare LC-MS vs. aptamer-based measurements (Figure 6D); as a dot plot to stratify positives and negatives based on the aptamer-based measurement and using the LC-MS data as the standard (Figure 6E); in the form of a Bangdiwala agreement chart, using an empirical threshold (based on clinical measurements) of 96 ng/mL of FTC levels to stratify positives vs. negatives; and in the form of an ROC curve (Figure S19). (5) Figure assembly was carried out using Adobe Illustrator. The Bandiwala chart was built using R code as indicated in the original reference (*BMC Med Res Methodol* **13**, 97 (2013)). The ROC curve was generated in Matlab using the ROC curve function.

# Table S1: DNA sequences used in this study.

| **Name** | **Sequence** |
| --- | --- |
| Bio-Cap_1 | 5′ – GTC GTC CCG AGA GCC ATA -BioTEG-3′ |
| Fwd_1 | 5′ – GGA GGC TCT CGG GAC GAC – 3′ |
| Rev_1 | 5′ -BioTEG- TTA CGA TTG CAG CAT CGG GAC G – 3′ |
| FTC_1_Full | 5′ – GGA GGC TCT CGG GAC GAC GCT GAG TCG TTG GCA AGG TAT TGA GAG AGT GTC GTC CCG ATG CTG CAA TCG TAA – 3′ |
| FTC_1_5’trunc8_3’trunc14 | 5′ – T CGG GAC GAC GCT GAG TCG TTG GCA AGG TAT TGA GAG AGT GTC GTC CCG A – 3′ |
| FTC_1_5’trunc15_3’trunc21 | 5′ – GAC ACT GAG TCG TTG GCA AGG TAT TGA GAG AGT GTC – 3′ |
| FTC_1_5’trunc16_3’trunc22 | 5′ – ACA CTG AGT CGT TGG CAA GGT ATT GAG AGA GTG T – 3′ |
| FTC_1_5’trunc17_3’trunc23 | 5′ – CAC TGA GTC GTT GGC AAG GTA TTG AGA GAG TG – 3′ |
| FTC_1_5’trunc18_3’trunc24 | 5′ – ACT GAG TCG TTG GCA AGG TAT TGA GAG AGT – 3′ |
| FTC_1_5’trunc19_3’trunc25  ***FTC_1_EAB*** | 5′ – CTG AGT CGT TGG CAA GGT ATT GAG AGA G – 3′ |
| FTC_1_5’trunc3_3’trunc14  *******FTC_1_Fluor*** | 5′ – /Cy5/-GGC TCT CGG GAC GAC GCT GAG TCG TTG GCA AGG TAT TGA GAG AGT GTC GTC CCG A – 3′ |
| Q-Cap_1 | 5′ – GTC GTC CCG AGA GCC-/BHQ_2_/ – 3′ |
| FTC_2_Full | 5′ – GGA GGC TCT CGG GAC GAC CAT GAG TCG TTG GTA AGG TAT TAG GAA GCG GTC GTC CCG ATG CTG CAA TCG TAA - 3′ |
| FTC_3_Full | 5′ – GGA GGC TCT CGG GAC GAC GGT CAG TCG TGG TAA GGT ATT ATG TGT AAC GTC GTC CCG ATG CTG CAA TCG TAA – 3′ |
| FTC_4_Full | 5′ – GGA GGC TCT CGG GAC GAC GTC AGT CGT TGG TAA GGT ATT AGT GTA ACG TCG TCC CGA TGC TGC AAT CGT AA – 3′ |
| FTC_5_Full | 5′ – GGA GGC TCT CGG GAC GAC GGC AAG TCG TTG GTA AGG TAT TAA TCT GGT GTC GTC CCG ATG CTG CAA TCG TAA – 3′ |
| FTC_1_EAB_Scramble | 5′ –GAT ATG GGA GAA CGC ATT GCG TGG TAT G – 3′ |

All sequences were synthesized following the above protocol. All surface constructs were modified on their 5’ end with hexanethiol and on their 3’ end with methylene blue.

Table S2: Detailed conditions used for FTC aptamer in vitro selection. Q = quick wash; R = repeat times.

| Rounds | 1 | 2 | 3 | 4 | 5 | 6 | 7 | 8 | 9 | 10 | 11 |
| --- | --- | --- | --- | --- | --- | --- | --- | --- | --- | --- | --- |
| C_Lib._ (μM) | 4 | 0.4 | 0.4 | 0.4 | 0.4 | 0.4 | 0.4 | 0.4 | 0.4 | 0.4 | 0.4 |
| n_Lib_ (pmol) | 1000 | 100 | 100 | 100 | 100 | 100 | 100 | 100 | 100 | 100 | 100 |
| C_Bio_C._ (μM) | 20 | 2 | 2 | 2 | 2 | 2 | 2 | 2 | 2 | 2 | 2 |
| n_Bio_C_(pmol) | 5000 | 500 | 500 | 500 | 500 | 500 | 500 | 500 | 500 | 500 | 500 |
| V_Lib&Bio_C_ (μL) | 250 | 250 | 250 | 250 | 250 | 250 | 250 | 250 | 250 | 250 | 250 |
| V_wash_ (mL) | 0.25 | 0.25 | 1 | 1 | 1 | 1 | 1 | 1 | 1 | 1 | 1 |
| T_wash_ (mins) | Q | Q | 1 | 1 | 1 | 1 | 1 | 1 | 1 | 1 | 1 |
| R_wash_(times) | 10 | 10 | 10 | 10 | 10 | 10 | 10 | 10 | 10 | 10 | 10 |
| C_dC._ (μM) | - | - | - | - | - | - | - | - | 50 | 50 | 50 |
| V_dC_ (mL) | - | - | - | - | - | - | - | - | 1 | 1 | 1 |
| T_dC_ (mins) | - | - | - | - | - | - | - | - | 1 | 30 | 30 |
| R_dC_ (times) | - | - | - | - | - | - | - | - | 3 | 3 | 3 |
| V_NSW_ (mL) | - | - | - | - | - | - | - | - | 1 | 1 | 1 |
| T_NSW_ (mins) | - | - | - | - | - | - | - | - | 1 | 1 | 1 |
| R_NSW_(times) | - | - | - | - | - | - | - | - | 3 | 3 | 3 |
| C_FTC._ (μM) | 100 | 100 | 100 | 50 | 50 | 25 | 25 | 10 | 10 | 1 | 1 |
| V_FTC_ (mL) | 0.25 | 0.25 | 0.25 | 0.25 | 0.25 | 0.25 | 0.25 | 0.25 | 0.25 | 0.25 | 0.25 |
| T_FTC_ (mins) | Q | Q | 1 | 1 | 1 | 1 | 1 | 1 | 1 | 1 | 1 |
| R_FTC_ (times) | 3 | 3 | 3 | 3 | 3 | 3 | 3 | 3 | 3 | 3 | 3 |
| Temp. (^o^C) | 23 | 23 | 23 | 23 | 23 | 23 | 23 | 23 | 23 | 23 | 23 |
| C_Mg_^2+^_._(mM) | 2 | 2 | 2 | 2 | 2 | 2 | 2 | 2 | 2 | 2 | 2 |

Notes:

1. The 1^st^ block (light orange) shows the library and Bio_cap strand mixture conditions from round 1 to 11.
2. The 2^nd^ block (light gold) shows the wash conditions from round 1 to 11.
3. The 3^rd^ block (light blue) shows the negative/counter selection conditions from round 1 to 11.
4. The 4^th^ block (light green) shows the conditions of extra wash after negative selection from round 1 to 11.
5. The 5^th^ block (light blue-grey) shows the positive selection conditions from round 1 to 11.
6. The 6^th^ block (light grey) shows other selection conditions from round 1 to 11.

Table S3: Lateral comparison of measured sample concentrations.

| Study | Participant ID | Visit | Aliquot Type | **CUID** | Position | FTC(ng/mL) Fluor | error | FTC ng/mL (LC/MS) |
| --- | --- | --- | --- | --- | --- | --- | --- | --- |
| Darunivir | GD02 | V2 | Plasma | **ZZX7WYA1** | 1 | N.D. | N.D. | <LOQ |
| Darunivir | GD01 | V2 | Plasma | **ZZX7WY9J** | 2 | N.D. | N.D. | <LOQ |
| Darunivir | GD04 | V2 | Plasma | **ZZX7WYB1** | 3 | N.D. | N.D. | <LOQ |
| Darunivir | GD09 | V2 | Plasma | **ZZX7WYDJ** | 4 | 839.56797 | 45.45448 | 816 |
| Darunivir | GD09 | V3 | Plasma | **ZZX7WXQJ** | 5 | N.D. | N.D. | 17 |
| Darunivir | GD07 | V2 | Plasma | **ZZX7WYCJ** | 6 | N.D. | N.D. | <LOQ |
| Darunivir | GD09 | V4 | Plasma | **ZZX7WX4V** | 7 | N.D. | N.D. | <LOQ |
| Darunivir | GD10 | V2 | Plasma | **ZZX7WYE1** | 8 | 889.7662 | 11.08678 | 587 |
| Darunivir | GD10 | V3 | Plasma | **ZZX7WXQV** | 9 | N.D. | N.D. | 47 |
| Darunivir | GD10 | V4 | Plasma | **ZZX7WX57** | 10 | N.D. | N.D. | 14 |
| Darunivir | GD11 | V2 | Plasma | **ZZX7WYEJ** | 11 | 1273.4227 | 44.83026 | 1310 |
| Darunivir | GD12 | V2 | Plasma | **ZZX7WYF1** | 12 | 1509.2324 | 36.43505 | 1345 |
| Darunivir | GD08 | V2 | Plasma | **ZZX7WYD1** | 13 | N.D. | N.D. | <LOQ |
| Darunivir | GD11 | V3 | Plasma | **ZZX7WXR7** | 14 | N.D. | N.D. | 36 |
| Darunivir | GD12 | V3 | Plasma | **ZZX7WXRJ** | 15 | N.D. | N.D. | 74 |
| Darunivir | GD11 | V4 | Plasma | **ZZX7WX5J** | 16 | N.D. | N.D. | <LOQ |
| Darunivir | GD12 | V4 | Plasma | **ZZX7WX5V** | 17 | N.D. | N.D. | <LOQ |
| Darunivir | GD13 | V2 | Plasma | **ZZX7WYFG** | 18 | 1978.9972 | 27.25647 | 1695 |
| Darunivir | GD17 | V2 | Plasma | **ZZX7WYHD** | 19 | 1218.8396 | 18.5696 | 867 |
| Darunivir | GD13 | V3 | Plasma | **ZZX7WXRV** | 20 | N.D. | N.D. | 32 |
| Darunivir | GD17 | V3 | Plasma | **ZZX7WXTD** | 21 | N.D. | N.D. | 24 |
| Darunivir | GD13 | V4 | Plasma | **ZZX7WX67** | 22 | N.D. | N.D. | <LOQ |
| Darunivir | GD17 | V4 | Plasma | **ZZX7WX7J** | 23 | N.D. | N.D. | <LOQ |
| Darunivir | GD20 | V2 | Plasma | **ZZX7WYID** | 24 | 1246.731 | 3.473482 | 1273 |
| Darunivir | GD20 | V3 | Plasma | **ZZX7WXUV** | 25 | N.D. | N.D. | 43 |
| Darunivir | GD23 | V2 | Plasma | **ZZX7WYJD** | 26 | 1575.3115 | 23.2573 | 1640 |
| Darunivir | GD24 | V2 | Plasma | **ZZX7WYJP** | 27 | 1138.6911 | 25.51348 | 1288 |
| Darunivir | GD23 | V3 | Plasma | **ZZX7WXWD** | 28 | N.D. | N.D. | 49 |
| Darunivir | GD24 | V3 | Plasma | **ZZX7WXWP** | 29 | N.D. | N.D. | 47 |
| Darunivir | GD23 | V4 | Plasma | **ZZX7WX9J** | 30 | N.D. | N.D. | <LOQ |
| Darunivir | GD24 | V4 | Plasma | **ZZX7WXA1** | 31 | N.D. | N.D. | <LOQ |
| Darunivir | GD25 | V2 | Plasma | **ZZX7WYK1** | 32 | 1969.8771 | 63.61124 | 2198 |
| Darunivir | GD25 | V3 | Plasma | **ZZX7WXX1** | 33 | N.D. | N.D. | 51 |
| Darunivir | GD22 | V2 | Plasma | **ZZX7WYJ1** | 34 | 1023.8464 | 12.21596 | 1260 |
| Darunivir | GD22 | V3 | Plasma | **ZZX7WXVV** | 35 | N.D. | N.D. | 43 |
| Darunivir | GD25 | V4 | Plasma | **ZZX7WXAJ** | 36 | N.D. | N.D. | <LOQ |
| Darunivir | GD22 | V4 | Plasma | **ZZX7WX97** | 37 | N.D. | N.D. | <LOQ |
| Darunivir | GD26 | V2 | Plasma | **ZZX7WYKD** | 38 | 1027.6933 | 22.55992 | 1543 |
| Darunivir | GD26 | V3 | Plasma | **ZZX7WXXD** | 39 | N.D. | N.D. | 48 |
| Darunivir | GD26 | V4 | Plasma | **ZZX7WXB1** | 40 | N.D. | N.D. | 15 |
| Darunivir | GD30 | V2 | Plasma | **ZZX7WYLP** | 41 | 625.80146 | 2.821197 | 779 |
| Darunivir | GD59 | V2 | Plasma | **ZZX86HN0** | 42 | 970.07972 | 16.09684 | 1915 |
| Darunivir | GD60 | V2 | Plasma | **ZZX86HNI** | 43 | 1021.4559 | 51.64935 | 1176 |
| Darunivir | GD30 | V3 | Plasma | **ZZX7WXYP** | 44 | N.D. | N.D. | 32 |
| Darunivir | GD59 | V3 | Plasma | **ZZX86HS0** | 45 | N.D. | N.D. | 47 |
| Darunivir | GD60 | V3 | Plasma | **ZZX86HSI** | 46 | N.D. | N.D. | 65 |
| Darunivir | GD30 | V4 | Plasma | **ZZX7WXD1** | 47 | N.D. | N.D. | <LOQ |
| Darunivir | GD59 | V4 | Plasma | **ZZX86HX0** | 48 | N.D. | N.D. | <LOQ |
| Darunivir | GD60 | V4 | Plasma | **ZZX86HXI** | 49 | N.D. | N.D. | <LOQ |
| Darunivir | GD31 | V2 | Plasma | **ZZX7WYM7** | 50 | 992.01007 | 25.82835 | 1503 |
| Darunivir | GD31 | V3 | Plasma | **ZZX7WXZ1** | 51 | N.D. | N.D. | 24 |
| Darunivir | GD31 | V4 | Plasma | **ZZX7WXDD** | 52 | N.D. | N.D. | <LOQ |
| Darunivir | GD32 | V2 | Plasma | **ZZX7WYMP** | 53 | 762.61038 | 8.894008 | 1030 |
| Darunivir | GD32 | V3 | Plasma | **ZZX7WXZD** | 54 | N.D. | N.D. | 19 |
| Darunivir | GD32 | V4 | Plasma | **ZZX7WXDP** | 55 | N.D. | N.D. | <LOQ |
| Darunivir | GD27 | V2 | Plasma | **ZZX7WYKP** | 56 | 931.59136 | 11.26914 | 1100 |
| Darunivir | GD27 | V3 | Plasma | **ZZX7WXXP** | 57 | N.D. | N.D. | 70 |
| Darunivir | GD27 | V4 | Plasma | **ZZX7WXBJ** | 58 | N.D. | N.D. | 15 |
| Darunivir | GD37 | V2 | Plasma | **ZZX7WYP7** | 59 | 543.70991 | 10.96834 | 888 |
| Darunivir | GD37 | V3 | Plasma | **ZZX7WY11** | 60 | N.D. | N.D. | 20 |
| Darunivir | GD37 | V4 | Plasma | **ZZX7WXFD** | 61 | N.D. | N.D. | <LOQ |
| Darunivir | GD39 | V2 | Plasma | **ZZX7WYPV** | 62 | 764.7619 | 20.23395 | 1117 |
| Darunivir | GD39 | V3 | Plasma | **ZZX7WY21** | 63 | N.D. | N.D. | 19 |
| Darunivir | GD39 | V4 | Plasma | **ZZX7WXG1** | 64 | N.D. | N.D. | <LOQ |
| Darunivir | GD40 | V2 | Plasma | **ZZX7WYQ7** | 65 | 426.73032 | 11.36284 | 694 |
| Darunivir | GD63 | V2 | Plasma | **ZZX86HP0** | 66 | 517.80963 | 19.85153 | 988 |
| Darunivir | GD40 | V3 | Plasma | **ZZX7WY2J** | 67 | N.D. | N.D. | 18 |
| Darunivir | GD63 | V3 | Plasma | **ZZX86HU0** | 68 | N.D. | N.D. | <LOQ |
| Darunivir | GD40 | V4 | Plasma | **ZZX7WXGD** | 69 | N.D. | N.D. | <LOQ |
| Darunivir | GD63 | V4 | Plasma | **ZZX86HZ0** | 70 | N.D. | N.D. | <LOQ |
| Darunivir | GD41 | V2 | Plasma | **ZZX7WYQJ** | 71 | 421.14212 | 21.78053 | 647 |
| Darunivir | GD44 | V2 | Plasma | **ZZX7WYRJ** | 72 | 506.47293 | 26.06003 | 1091 |
| Darunivir | GD41 | V3 | Plasma | **ZZX7WY31** | 73 | N.D. | N.D. | 26 |
| Darunivir | GD44 | V3 | Plasma | **ZZX7WY4J** | 74 | N.D. | N.D. | <LOQ |
| Darunivir | GD41 | V4 | Plasma | **ZZX7WXGP** | 75 | N.D. | N.D. | <LOQ |
| Darunivir | GD44 | V4 | Plasma | **ZZX7WXHP** | 76 | N.D. | N.D. | <LOQ |
| Darunivir | GD47 | V2 | Plasma | **ZZX7WYSJ** | 77 | 358.76039 | 4.582827 | 485 |
| Darunivir | GD47 | V3 | Plasma | **ZZX7WY5J** | 78 | N.D. | N.D. | 31 |
| Darunivir | GD47 | V4 | Plasma | **ZZX7WXJ7** | 79 | N.D. | N.D. | <LOQ |
| Darunivir | GD67 | V2 | Plasma | **ZZX86HR0** | 80 | 810.21722 | 8.324475 | 676 |
| Darunivir | GD67 | V3 | Plasma | **ZZX86HW0** | 81 | N.D. | N.D. | 25 |
| Darunivir | GD67 | V4 | Plasma | **ZZX86I10** | 1 | N.D. | N.D. | <LOQ |
| Darunivir | GD48 | V2 | Plasma | **ZZX7WYSV** | 2 | 1475.1065 | 30.53448 | 799 |
| Darunivir | GD69 | V2 | Plasma | **ZZX8J1RE** | 3 | 1078.859 | 32.21807 | 414 |
| Darunivir | GD48 | V3 | Plasma | **ZZX7WY5V** | 4 | N.D. | N.D. | 16 |
| Darunivir | GD69 | V3 | Plasma | **ZZX8J1WE** | 5 | N.D. | N.D. | <LOQ |
| Darunivir | GD48 | V4 | Plasma | **ZZX7WXJP** | 6 | N.D. | N.D. | <LOQ |
| Darunivir | GD69 | V4 | Plasma | **ZZX8J21E** | 7 | N.D. | N.D. | <LOQ |
| Darunivir | GD66 | V2 | Plasma | **ZZX86HQI** | 8 | 802.37209 | 20.52828 | 315 |
| Darunivir | GD68 | V2 | Plasma | **ZZX86HRI** | 9 | 1325.072 | 37.55446 | 554 |
| Darunivir | GD52 | V2 | Plasma | **ZZX7WYU7** | 10 | 178.91343 | 4.636875 | 142 |
| Darunivir | GD52 | V3 | Plasma | **ZZX7WY77** | 11 | N.D. | N.D. | 25 |
| Darunivir | GD66 | V3 | Plasma | **ZZX86HVI** | 12 | N.D. | N.D. | <LOQ |
| Darunivir | GD68 | V3 | Plasma | **ZZX86HWI** | 13 | N.D. | N.D. | <LOQ |
| Darunivir | GD53 | V2 | Plasma | **ZZX7WYUJ** | 14 | 12.544208 | 0.106603 | 96 |
| Darunivir | GD52 | V4 | Plasma | **ZZX7WXLJ** | 15 | N.D. | N.D. | <LOQ |
| Darunivir | GD53 | V3 | Plasma | **ZZX7WY7J** | 16 | N.D. | N.D. | <LOQ |
| Darunivir | GD53 | V4 | Plasma | **ZZX7WXLV** | 17 | N.D. | N.D. | <LOQ |
| Darunivir | GD68 | V4 | Plasma | **ZZX86I1I** | 18 | N.D. | N.D. | <LOQ |
| Darunivir | GD55 | V2 | Plasma | **ZZX7WYV7** | 19 | 1023.1049 | 13.44172 | 424 |
| Darunivir | GD55 | V3 | Plasma | **ZZX7WY87** | 20 | N.D. | N.D. | 53 |
| Darunivir | GD54 | V2 | Plasma | **ZZX7WYUV** | 21 | 128.19848 | 3.09697 | 99 |
| Darunivir | GD54 | V3 | Plasma | **ZZX7WY7V** | 22 | N.D. | N.D. | <LOQ |
| Darunivir | GD55 | V4 | Plasma | **ZZX7WXMJ** | 23 | N.D. | N.D. | 43 |
| Darunivir | GD57 | V2 | Plasma | **ZZX7WYVV** | 24 | 36.506943 | 1.057921 | 75 |
| Darunivir | GD54 | V4 | Plasma | **ZZX7WXM7** | 25 | N.D. | N.D. | <LOQ |
| Darunivir | GD57 | V3 | Plasma | **ZZX7WY8V** | 26 | N.D. | N.D. | <LOQ |
| Darunivir | GD57 | V4 | Plasma | **ZZX7WXN7** | 27 | N.D. | N.D. | <LOQ |
| Genvoya PEP | PP01 | V2 | Plasma | **ZZX8IGI3** | 28 | N.D. | N.D. | <LOQ |
| Genvoya PEP | PP02 | V2 | Plasma | **ZZX8IGIX** | 29 | N.D. | N.D. | <LOQ |
| Genvoya PEP | PP04 | V2 | Plasma | **ZZX8IGKL** | 30 | N.D. | N.D. | <LOQ |
| Genvoya PEP | PP07 | V4 | Plasma | **ZZX8IGN3** | 31 | 2347.8504 | 15.723 | 3315 |
| Genvoya PEP | PP08 | V4 | Plasma | **ZZX8IGNX** | 32 | 1739.6376 | 23.90075 | 2565 |
| Genvoya PEP | PP15 | V4 | Plasma | **ZZX8IGTL** | 33 | 2099.3721 | 11.85886 | 3310 |
| Genvoya PEP | PP08 | V5 | Plasma | **ZZX8IHRR** | 34 | N.D. | N.D. | <LOQ |
| Genvoya PEP | PP15 | V5 | Plasma | **ZZX8IHVT** | 35 | N.D. | N.D. | <LOQ |
| Genvoya PEP | PP09 | V4 | Plasma | **ZZX8IGOR** | 36 | 1182.3049 | 34.58373 | 1770 |
| Genvoya PEP | PP18 | V4 | Plasma | **ZZX8IGV9** | 37 | 2073.4842 | 22.54788 | 3345 |
| Genvoya PEP | PP09 | V5 | Plasma | **ZZX8IHSB** | 38 | N.D. | N.D. | <LOQ |
| Genvoya PEP | PP18 | V5 | Plasma | **ZZX8IHYB** | 39 | N.D. | N.D. | <LOQ |
| Genvoya PEP | PP12 | V4 | Plasma | **ZZX8IGR9** | 40 | 1437.6278 | 20.83014 | 2755 |
| Genvoya PEP | PP12 | V5 | Plasma | **ZZX8IHTZ** | 41 | N.D. | N.D. | <LOQ |
| Genvoya PEP | PP20 | V4 | Plasma | **ZZX8IGWD** | 42 | 1377.0701 | 47.34739 | 2665 |
| Genvoya PEP | PP20 | V5 | Plasma | **ZZX8IHZZ** | 43 | N.D. | N.D. | <LOQ |
| Genvoya PEP | PP24 | V4 | Plasma | **ZZX8IGZ1** | 44 | 956.59593 | 21.23845 | 1825 |
| Genvoya PEP | PP25 | V4 | Plasma | **ZZX8IGZV** | 45 | 1345.4418 | 28.76541 | 2565 |
| Genvoya PEP | PP23 | V4 | Plasma | **ZZX8IGY7** | 46 | 1041.1877 | 10.0305 | 1625 |
| Genvoya PEP | PP24 | V5 | Plasma | **ZZX8II2V** | 47 | N.D. | N.D. | <LOQ |
| Genvoya PEP | PP25 | V5 | Plasma | **ZZX8II3F** | 48 | N.D. | N.D. | <LOQ |
| Genvoya PEP | PP23 | V5 | Plasma | **ZZX8II2B** | 49 | N.D. | N.D. | <LOQ |
| Genvoya PEP | PP26 | V4 | Plasma | **ZZX8IH0P** | 50 | 1018.1433 | 12.77287 | 1585 |
| Genvoya PEP | PP31 | V4 | Plasma | **ZZX8IH4V** | 51 | 426.56668 | 15.06631 | 747 |
| Genvoya PEP | PP32 | V4 | Plasma | **ZZX8IH5J** | 52 | 1178.5687 | 30.12535 | 1860 |
| Genvoya PEP | PP26 | V5 | Plasma | **ZZX8II3Z** | 53 | N.D. | N.D. | <LOQ |
| Genvoya PEP | PP31 | V5 | Plasma | **ZZX8II6X** | 54 | N.D. | N.D. | <LOQ |
| Genvoya PEP | PP32 | V5 | Plasma | **ZZX8II7R** | 55 | N.D. | N.D. | <LOQ |
| Genvoya PEP | PP34 | V4 | Plasma | **ZZX8IH6N** | 56 | 1065.0557 | 6.889254 | 2435 |
| Genvoya PEP | PP33 | V4 | Plasma | **ZZX8IH63** | 57 | 912.76369 | 57.16135 | 1145 |
| Genvoya PEP | PP34 | V5 | Plasma | **ZZX8II9F** | 58 | N.D. | N.D. | <LOQ |
| Genvoya PEP | PP33 | V5 | Plasma | **ZZX8II8L** | 59 | N.D. | N.D. | <LOQ |
| Genvoya PEP | PP39 | V4 | Plasma | **ZZX8IH9L** | 60 | N.D. | N.D. | 178 |
| Genvoya PEP | PP39 | V5 | Plasma | **ZZX8IIDF** | 61 | N.D. | N.D. | <LOQ |
| Genvoya PEP | PP40 | V4 | Plasma | **ZZX8IHAF** | 62 | N.D. | N.D. | 102.5 |
| Genvoya PEP | PP40 | V5 | Plasma | **ZZX8IIDZ** | 63 | N.D. | N.D. | <LOQ |
| Genvoya PEP | PP22 | V4 | Plasma | **ZZX8IGXH** | 64 | 896.20457 | 30.47413 | 1765 |
| Genvoya PEP | PP41 | V4 | Plasma | **ZZX8IHB9** | 65 | N.D. | N.D. | 223.5 |
| Genvoya PEP | PP42 | V4 | Plasma | **ZZX8IHC3** | 66 | N.D. | N.D. | 257.5 |
| Genvoya PEP | PP22 | V5 | Plasma | **ZZX8II1N** | 67 | N.D. | N.D. | 767.5 |
| Genvoya PEP | PP41 | V5 | Plasma | **ZZX8IIEJ** | 68 | N.D. | N.D. | <LOQ |
| Genvoya PEP | PP42 | V5 | Plasma | **ZZX8IIF3** | 69 | N.D. | N.D. | <LOQ |
| Genvoya PEP | PP50 | V4 | Plasma | **ZZX8IHHR** | 70 | N.D. | N.D. | 124.25 |
| Genvoya PEP | PP50 | V5 | Plasma | **ZZX8IIKJ** | 71 | N.D. | N.D. | 107.025 |
| Genvoya PEP | PP55 | V4 | Plasma | **ZZX8IHKP** | 72 | 171.32143 | 9.428656 | 225 |
| Genvoya PEP | PP57 | V4 | Plasma | **ZZX8IHMD** | 73 | 251.50214 | 5.325073 | 350.5 |
| Genvoya PEP | PP51 | V4 | Plasma | **ZZX8IHIB** | 74 | N.D. | N.D. | 55.65 |
| Genvoya PEP | PP49 | V4 | Plasma | **ZZX8IHH7** | 75 | N.D. | N.D. | 37.025 |
| Genvoya PEP | PP59 | V4 | Plasma | **ZZX8IHO1** | 76 | 157.5492 | 5.314966 | 317 |
| Genvoya PEP | PP60 | V4 | Plasma | **ZZX8IHOV** | 77 | 110.94822 | 1.190456 | 282.5 |
| Genvoya PEP | PP51 | V5 | Plasma | **ZZX8IILD** | 78 | N.D. | N.D. | <LOQ |
| Genvoya PEP | PP49 | V5 | Plasma | **ZZX8IIJP** | 79 | N.D. | N.D. | <LOQ |
| Genvoya PEP | PP53 | V4 | Plasma | **ZZX8IHJF** | 80 | N.D. | N.D. | 64.525 |
| Genvoya PEP | PP53 | V5 | Plasma | **ZZX8IIN1** | 81 | N.D. | N.D. | <LOQ |

| 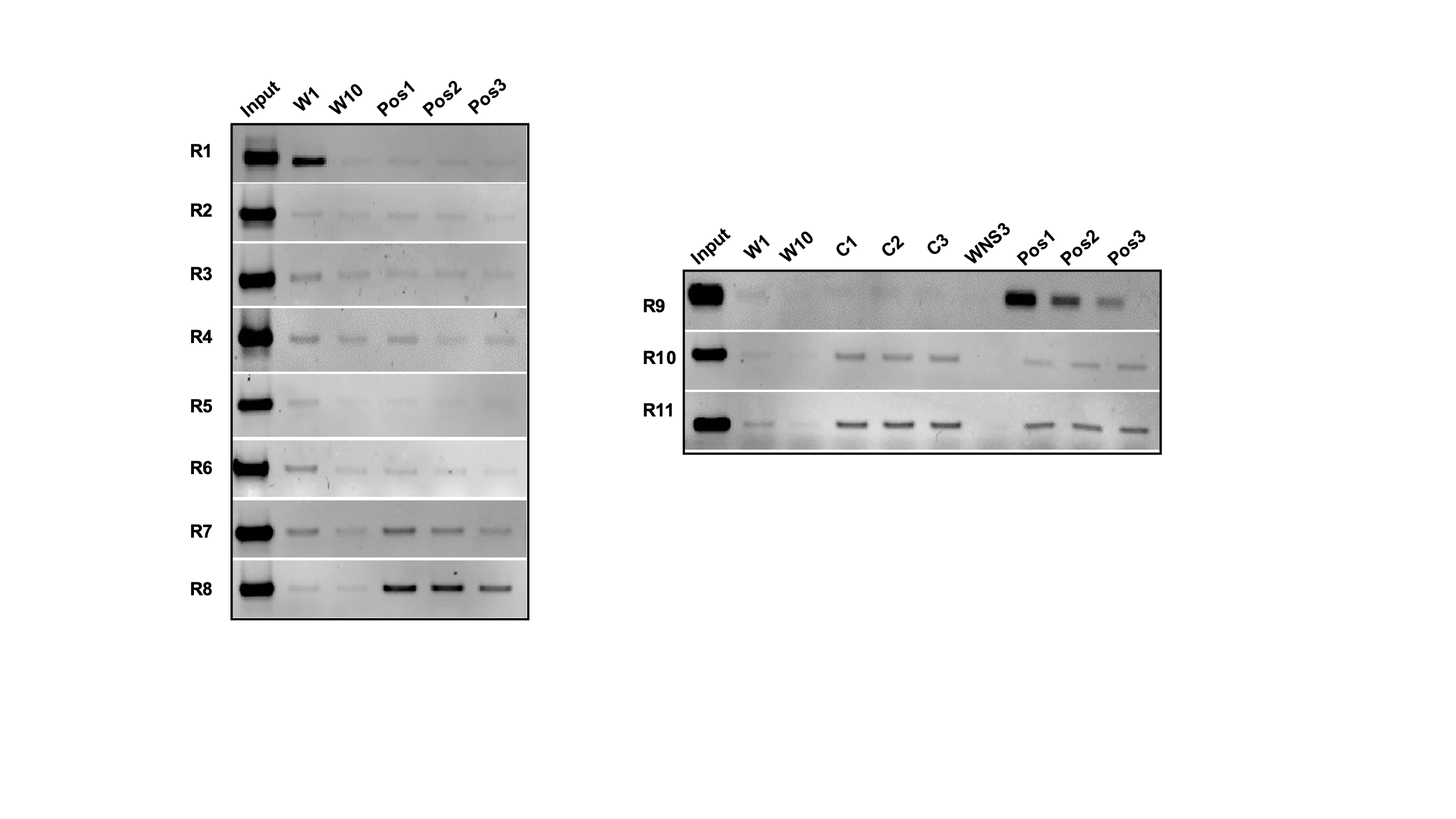 |
| --- |
| Fig S1: Monitoring capture-SELEX progress by elution profiles**.** Several eluents, including the library input (Input), 1^st^ and 10^th^ washing step prior to selection (W1 and W10, respectively), three counter selection washes with deoxycytidine (C1 – 3), and three positive selection washes with FTC (Pos1 – 3), were collected during the selection, amplified via small scale PCR, and analyzed by agarose gel. Round number (R) is indicated next to each row. The counter selection was introduced during round 9 and onward. |

| 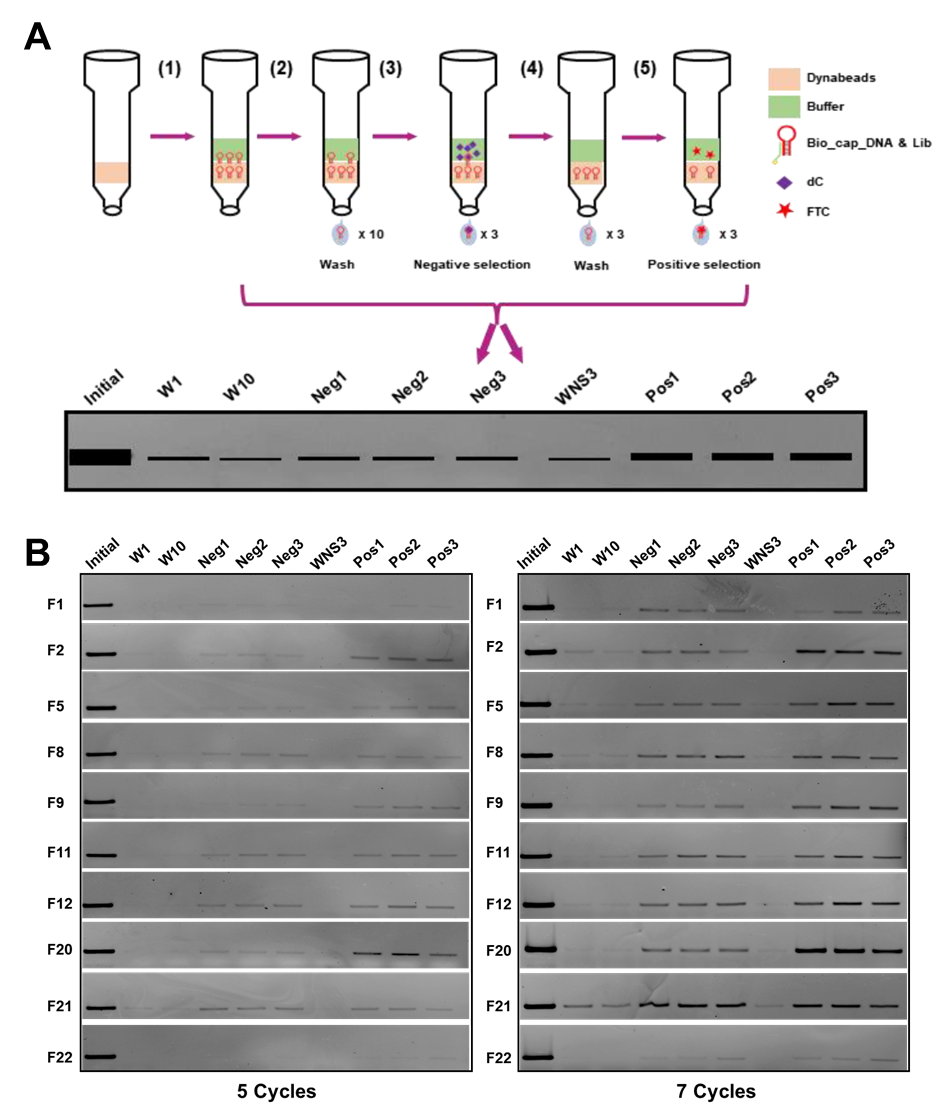 |
| --- |
| Fig S2: Clone SELEX elution profiles of 10 candidates. The elution profile of clone F20 (FTC_1_Full) is the best. (A) During selection, several fractions including the initial library, 1st and 10th wash, negative selection 1 to 3, 3rd time of additional wash, and positive selection 1 to 3, were collected to generate the elution profile. Illustration of selection procedure. (1) Attach the library to Dyna beads; (2) Wash the beads with SELEX buffer to remove weakly bound ssDNA molecules; (3) Negative/Counter selection, applied from round 9 to 11; (4) Additional wash to remove residual solution from the negative selection, applied from round 9 to 11; (5) Positive selection. (B) The elution profile of 10 candidates. The collected supernatants during the selection were compared though small-scale PCR. In the profile, the PCR product of initial library was utilized as both positive control and marker to verify product size. The elution profile was monitored at 5 cycles and 7 cycles of PCR. |

| 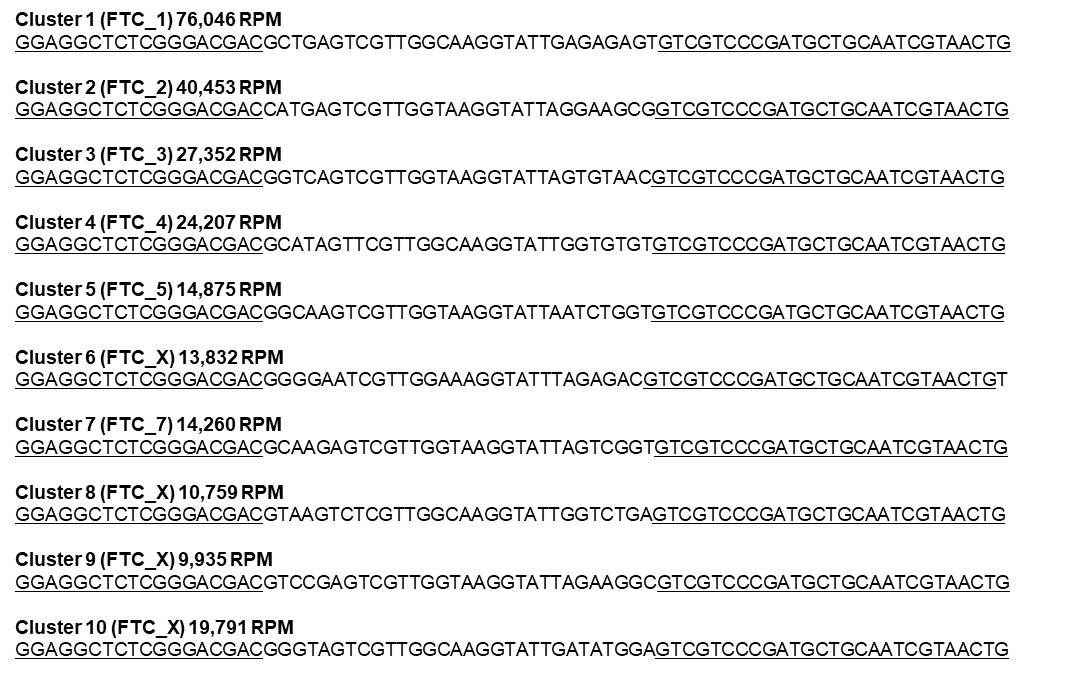 |
| --- |
| Fig S3: Sequencing results of round 11 pool from aptamer selection. The parent sequence from each cluster is shown. |

| 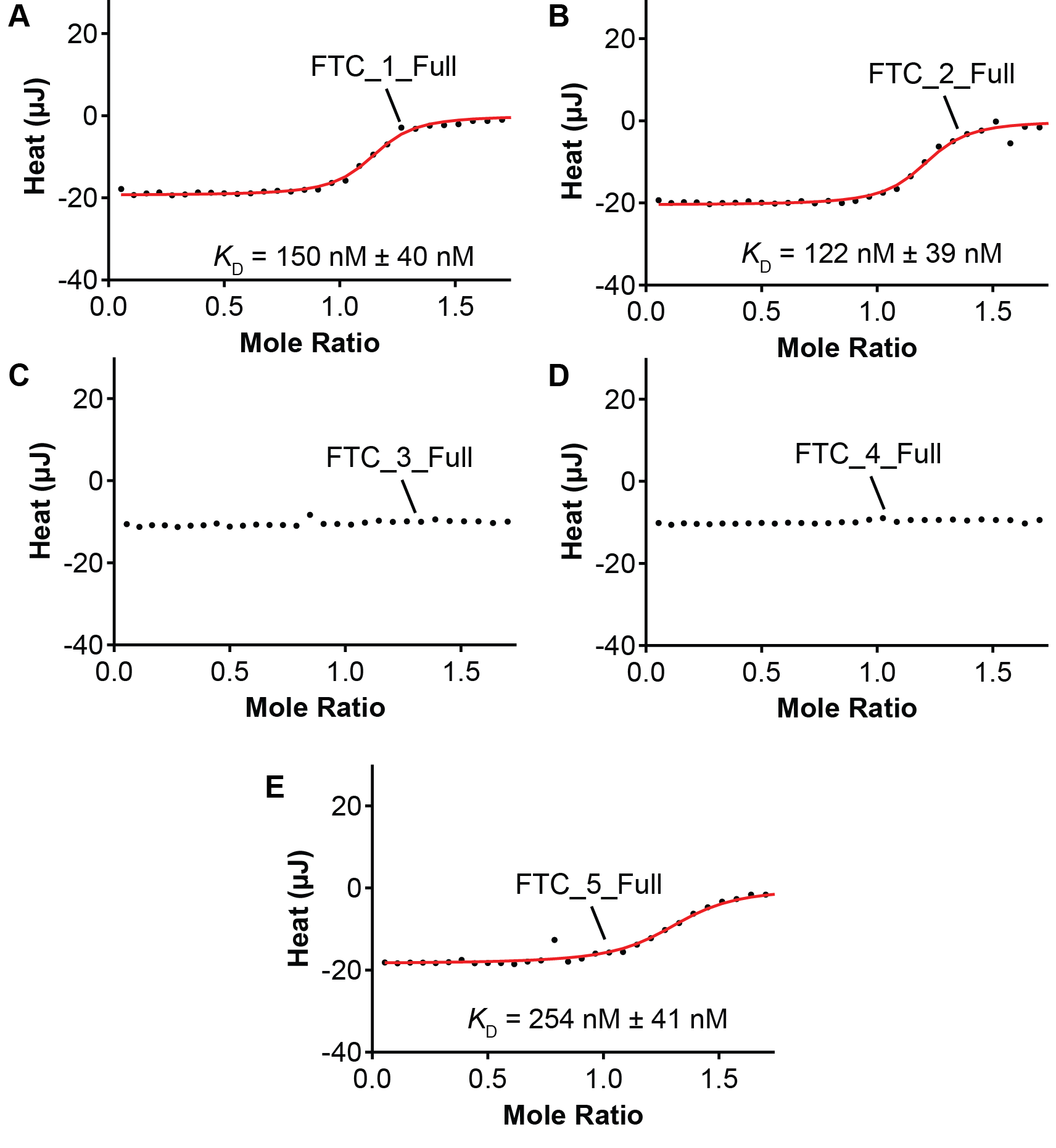 |
| --- |
| Fig S4: Binding of FTC to top cluster sequences via ITC. Clusters 1 (A) and 2 (B) display strong binding to FTC via isothermal titration calorimetry. Top sequences from clusters 3 (C) and 4 (D) display no apparent binding, but do display some interaction with the molecule, as shown by a negative heat exchange upon addition of target. (E) The top sequence form cluster 5 displays strong binding to FTC. |

| 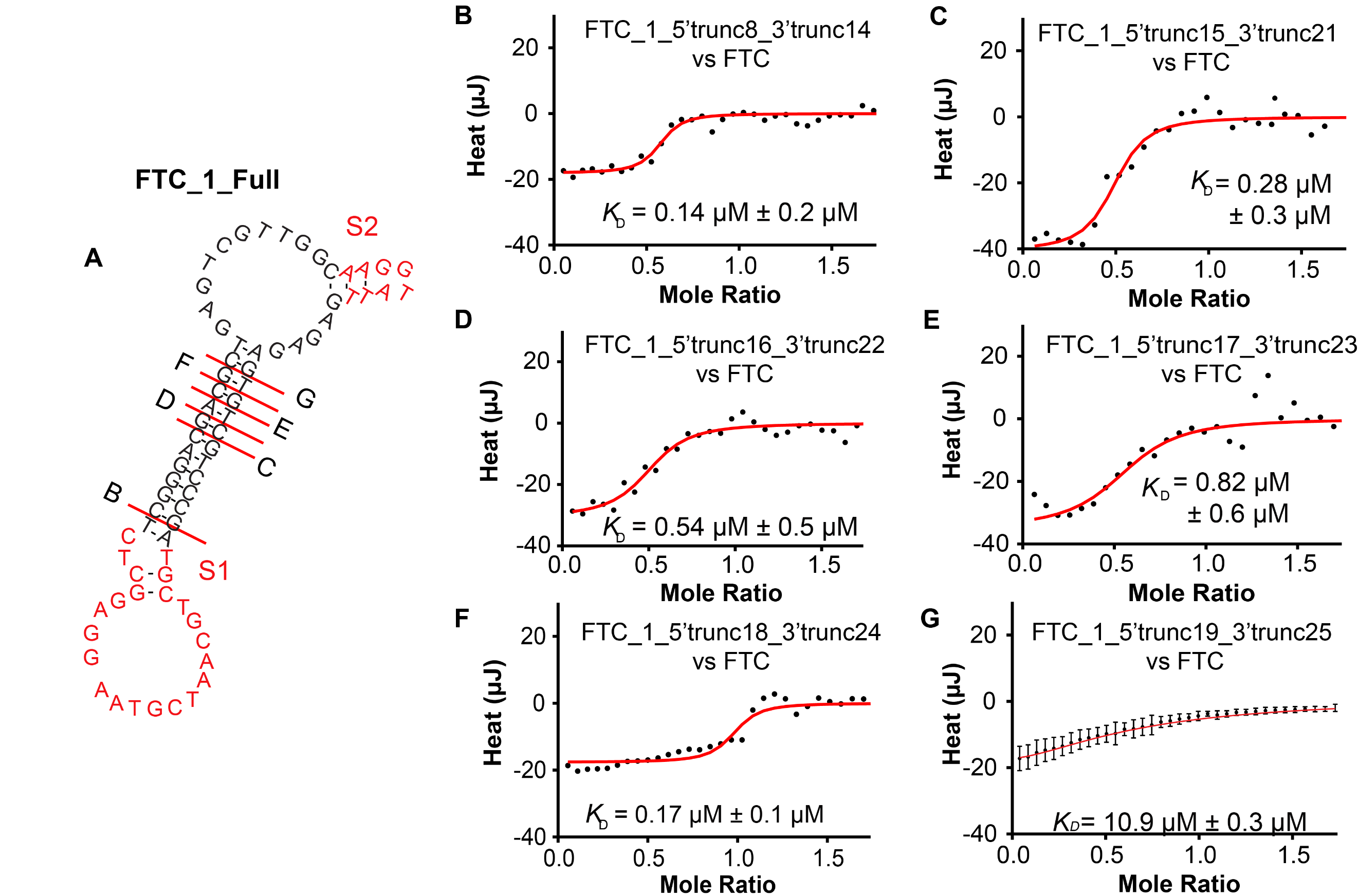 |
| --- |
| Fig S5: Serial truncations of FTC_1_Full maintain FTC binding. ITC thermograms showing affinity-based interaction between various truncations of FTC_1_Full and FTC. |

| 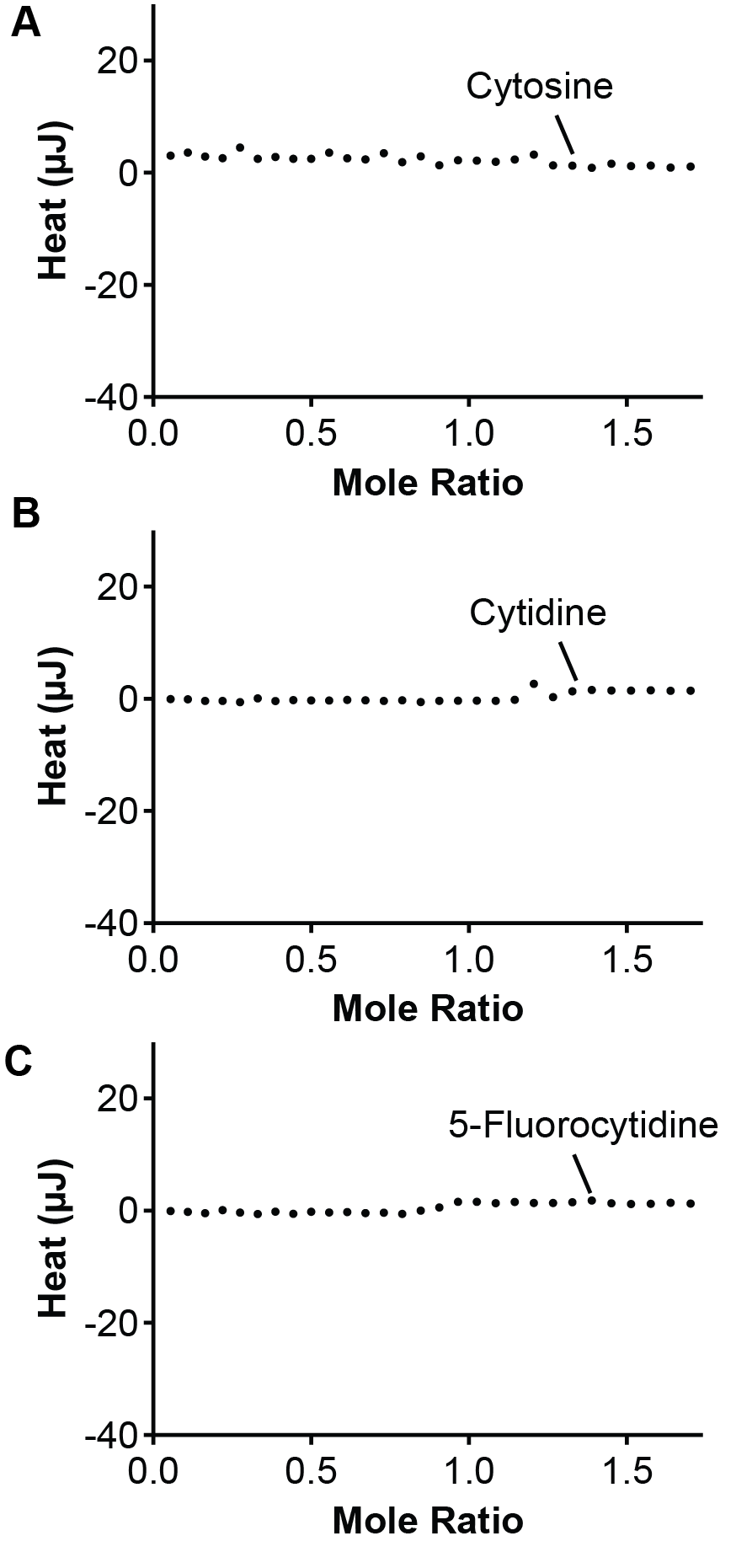 |
| --- |
| Fig S6: ITC thermograms of FTC_1_EAB against close analogs of FTC. When challenged with either cytosine (A), cytidine (B), or 5-Fluorocytidine (C), FTC_1_EAB displays no binding. |

| 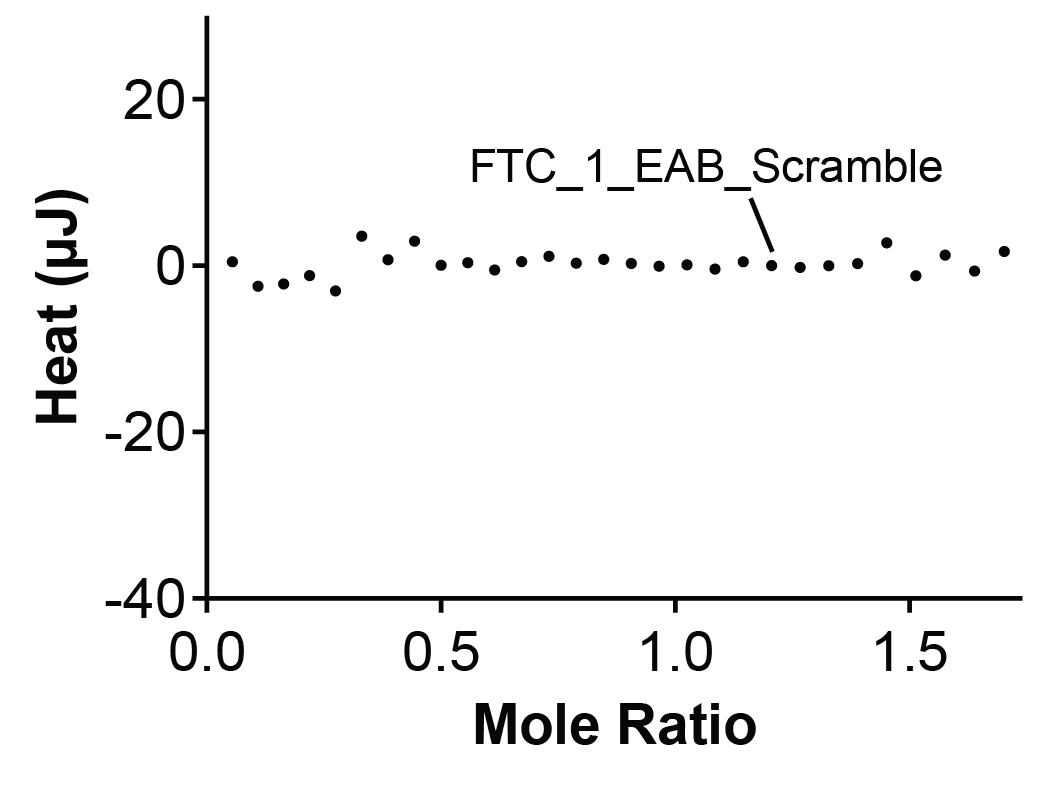 |
| --- |

Fig S7: A scrambled version of FTC_1_EAB displays no binding to FTC**.** Scrambled sequence FTC_1_EAB_Scramble found in Table S1.

| 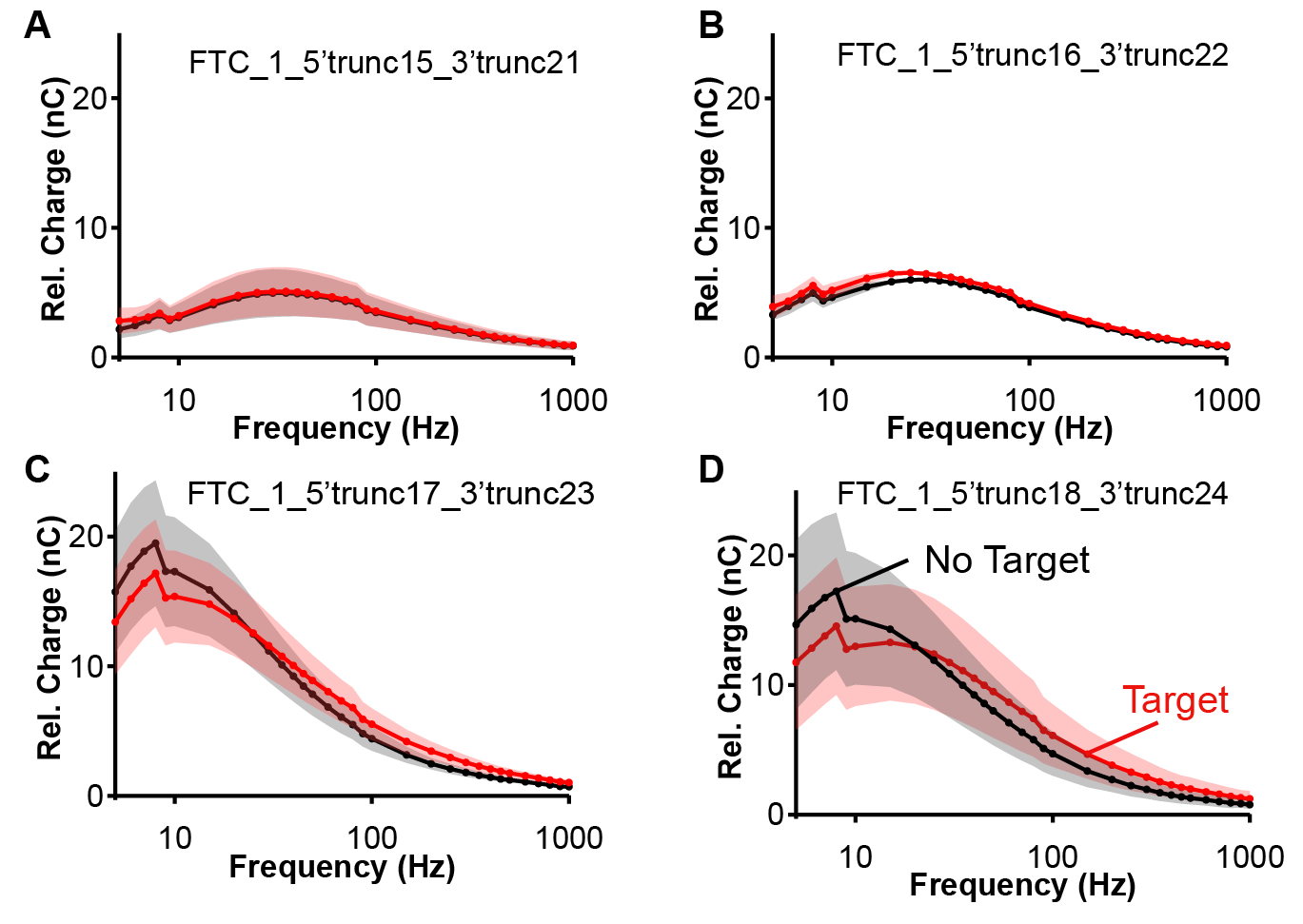 |
| --- |
| Fig S8: Frequency maps of FTC_1 truncations. FTC_1 truncations were modified with a hexanethiol linker at the 5’ end and with a methylene blue at the 3’ end. Sensors are interrogated in the absence (black trace) and presence (red trace) of 10 µM FTC resulting in frequency maps displaying relative charge at a given square wave frequency. The presence of a lateral shift along the x-axis signifies conformational switching of the surface bound aptamer similar to Fig. 4A. |

| 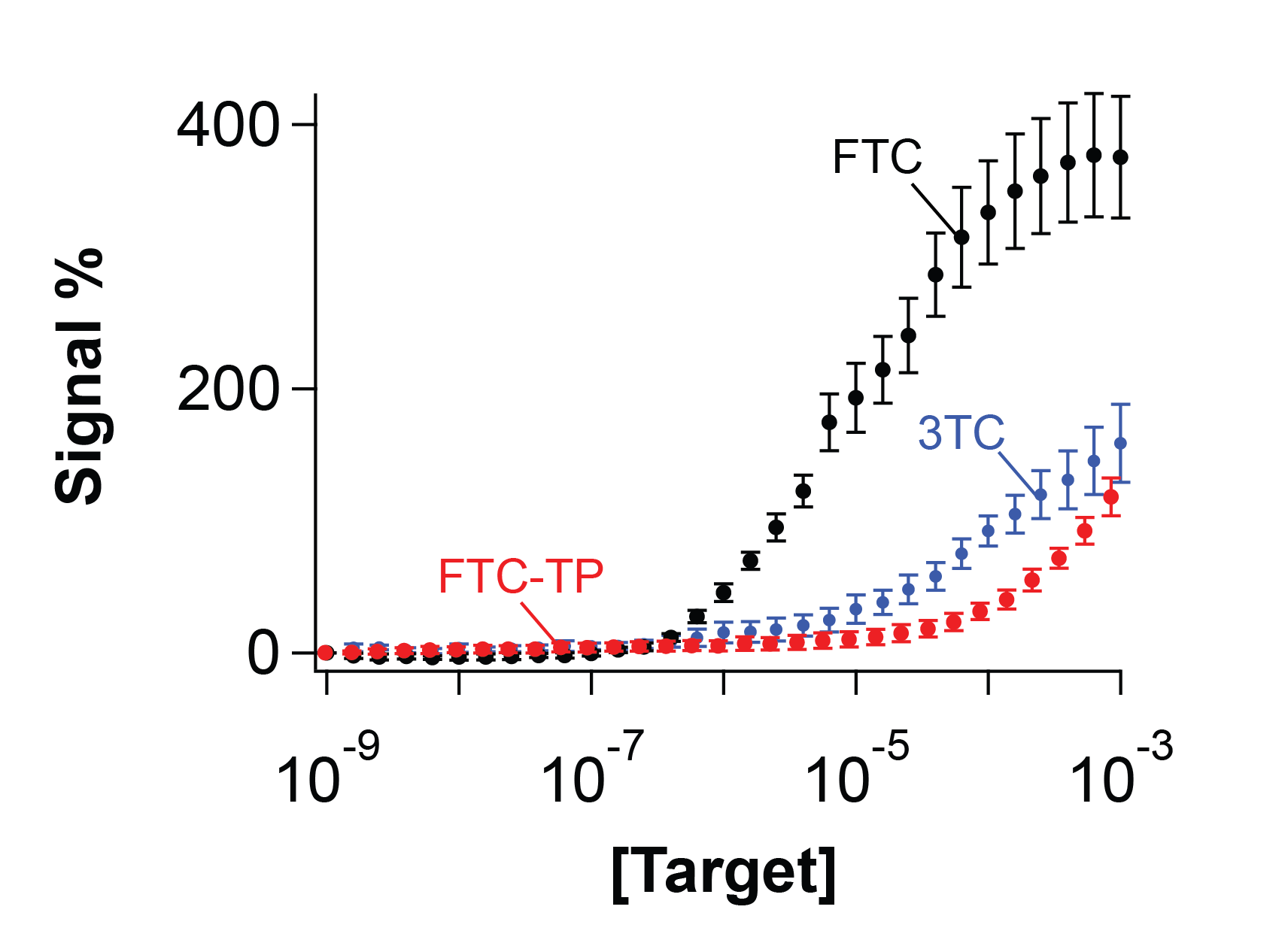 |
| --- |
| Fig S9: Dose response curves of FTC_1_EAB against structural analogs. FTC_1_EAB challenged with FTC (black trace) reveals maximum signal gain at ~400% with an EC50 of 10.4 µM ± 0.6 µM (Fig. 4D). When challenged with either lamivudine (3TC, blue trace) or FTC-triphosphate (FTC-TP, red trace), minimal binding is observed at saturation, with no significant signal change in physiologically relevant concentrations. |

| 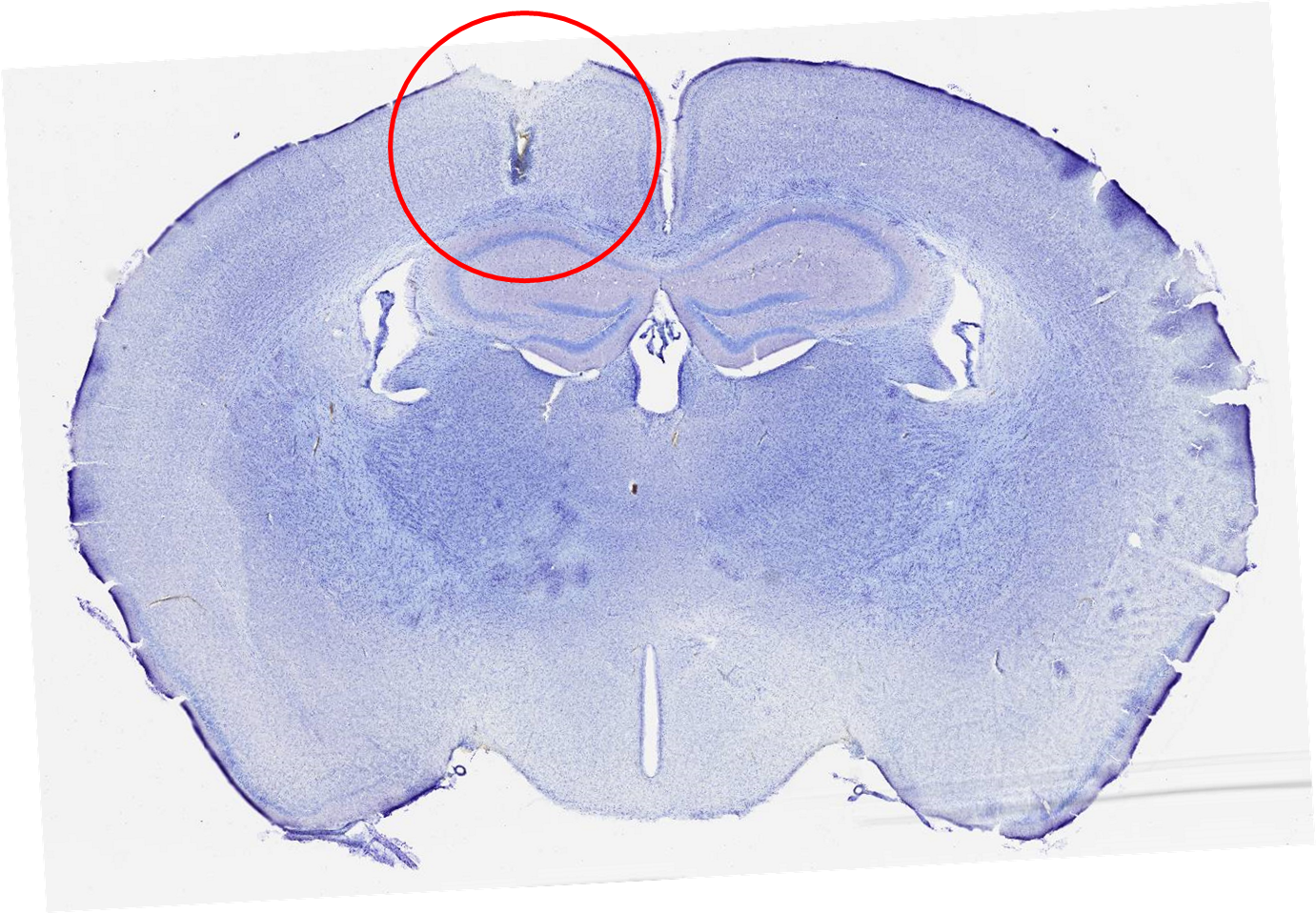 |
| --- |
| Fig S10: Representative histology image from in vivo brain experiments. Prior to animal euthanasia a 300 µA current was passed through the sensor for 2 s to locally burn the tissue, highlighting sensor placement in red circle. |

| 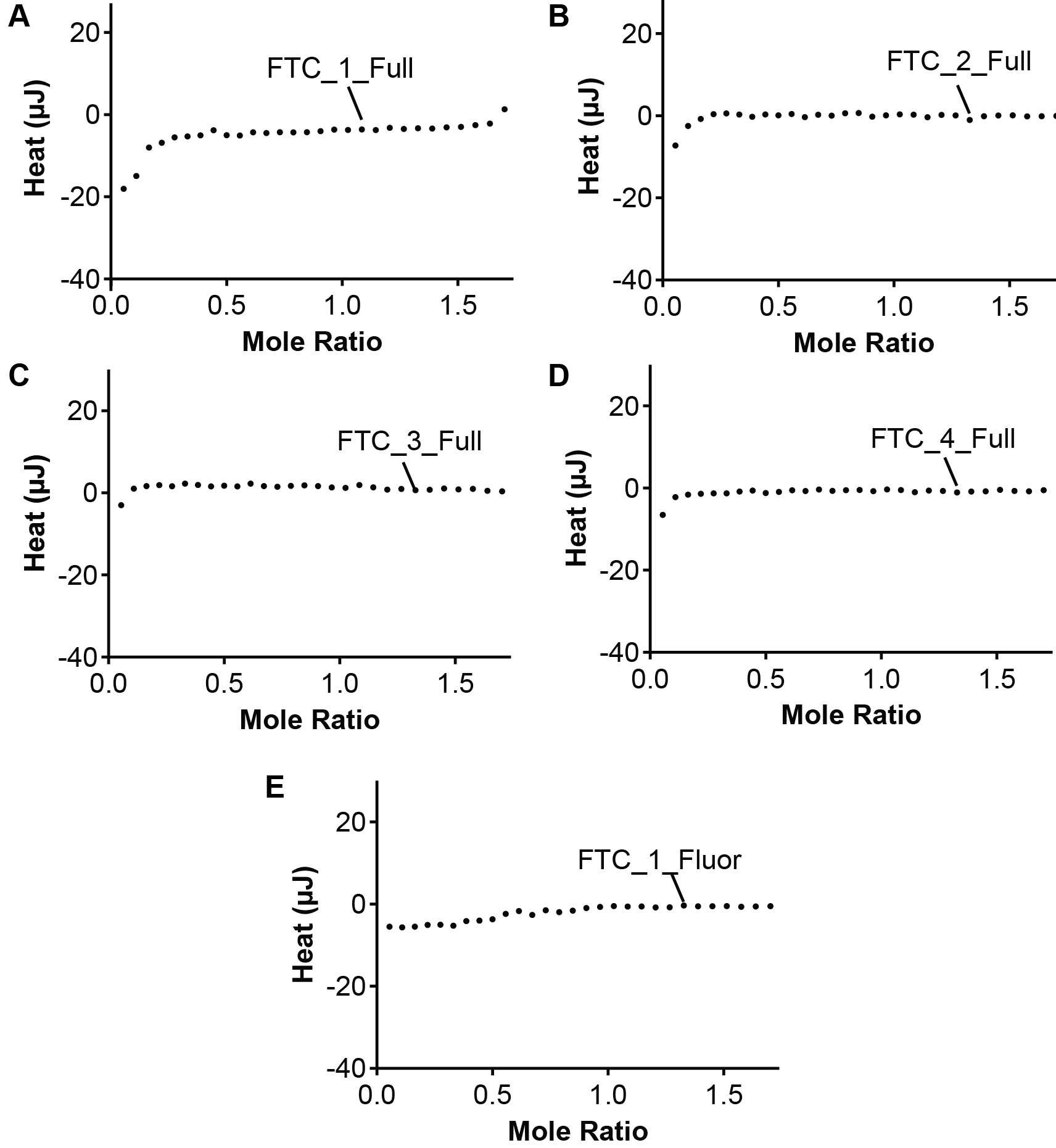 |
| --- |
| Fig S11: ITC thermograms of FTC binding aptamers against BSA**.** All sequences are found in Table S1. |

| 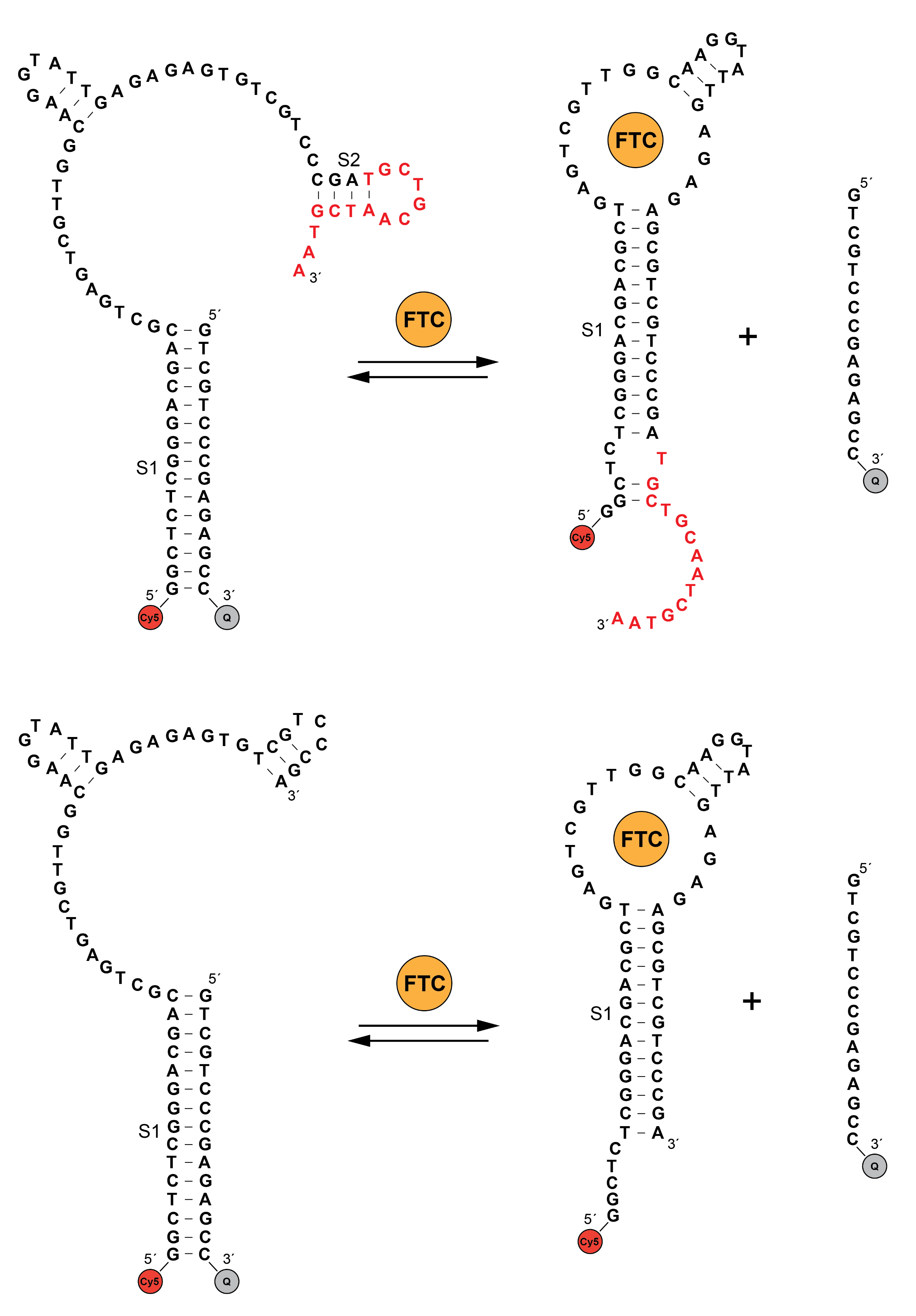 |
| --- |
| Fig S12: Mechanism of action of FTC_1_Fluor. Upper panels represent mechanism of action for FTC_1_5’trunc3. Lower panels represent mechanism of action of FTC_1_Fluor. |

| 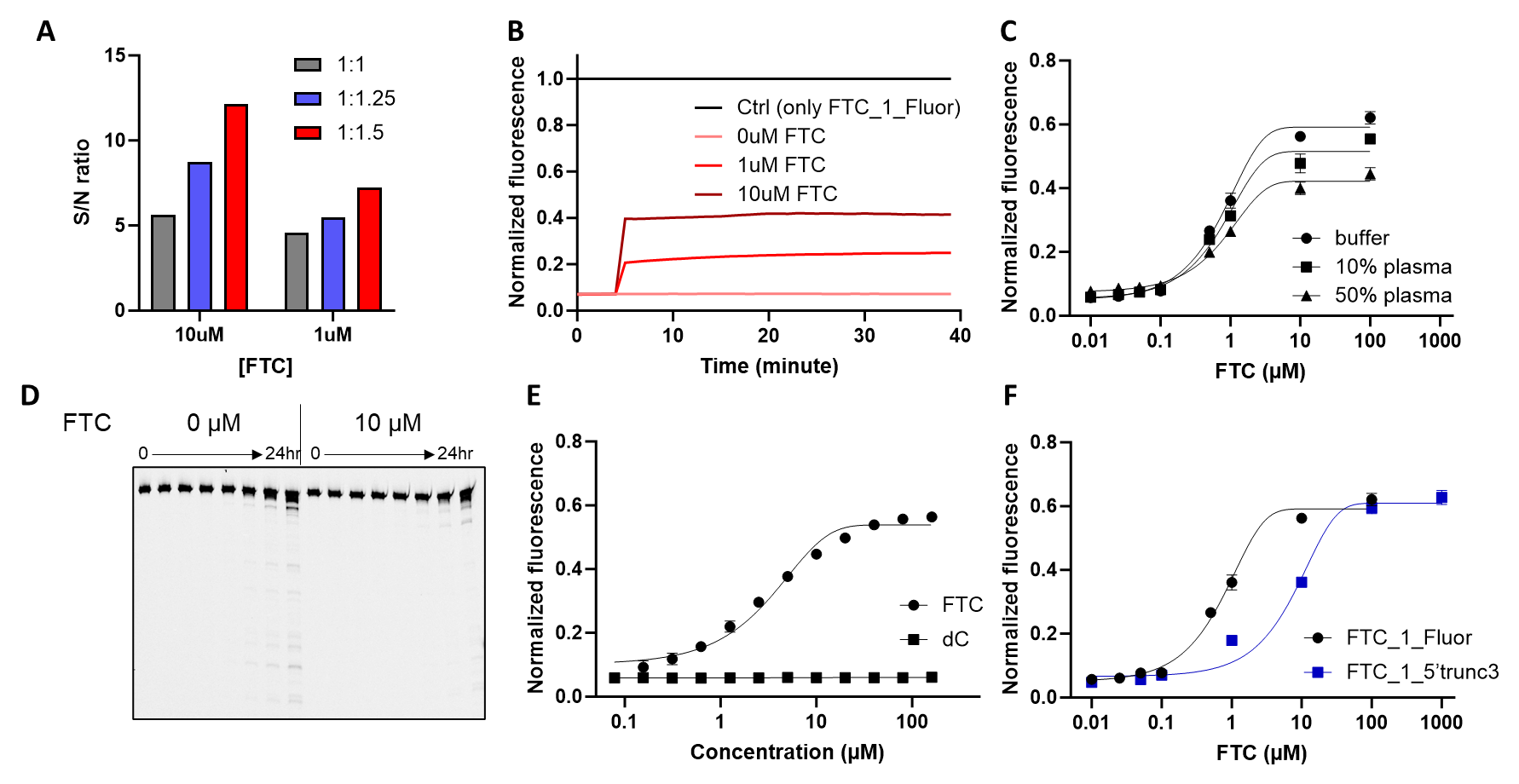 |
| --- |
| Fig S13: Normalized fluorescence of modified constructs. Truncation of 14 nucleotides from the 3′terminus of the FTC_1_5′trunc3 to eliminate unpaired nucleotides extending from stem S1, resulting in a rapid aptamer-based optical sensor. This truncation achieved optimal FTC detection across the clinically relevant range of 100 nM to 10 µM. |

| 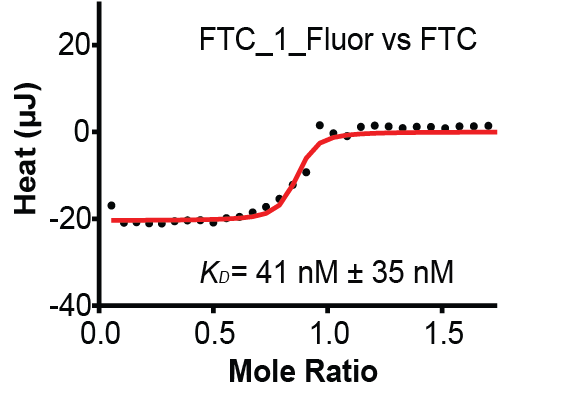 |
| --- |
| Fig S14: ITC Thermogram of FTC_1_Fluor against FTC. The truncated construct maintains similar binding affinity to the full-length construct (Fig. 3B). |

| 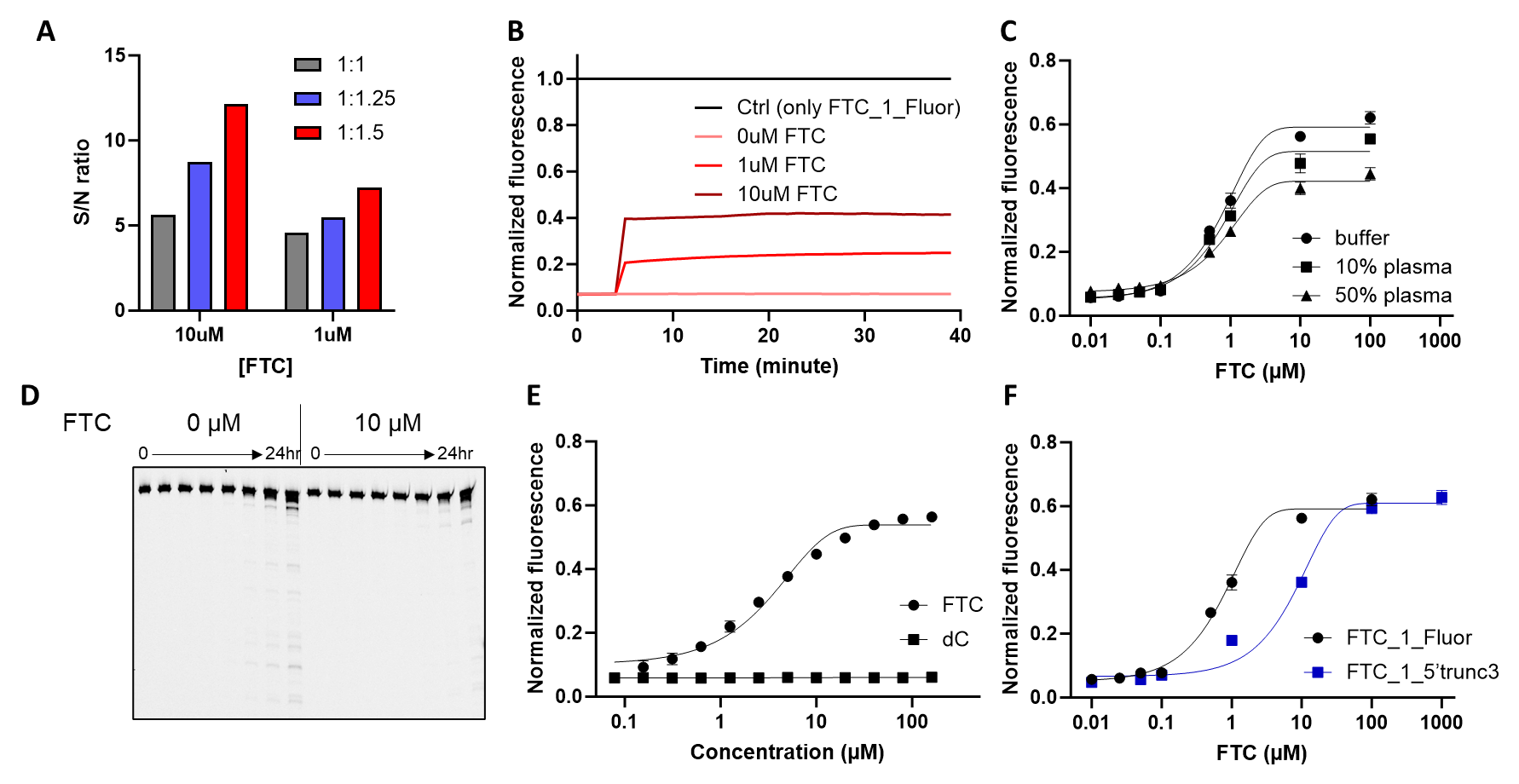 |
| --- |
| Fig S15: Specificity of FTC_1_Fluor against dC. No fluorescence signal was observed in the presence of excess dC, confirming the molecular specificity of the sensor for FTC. |

| 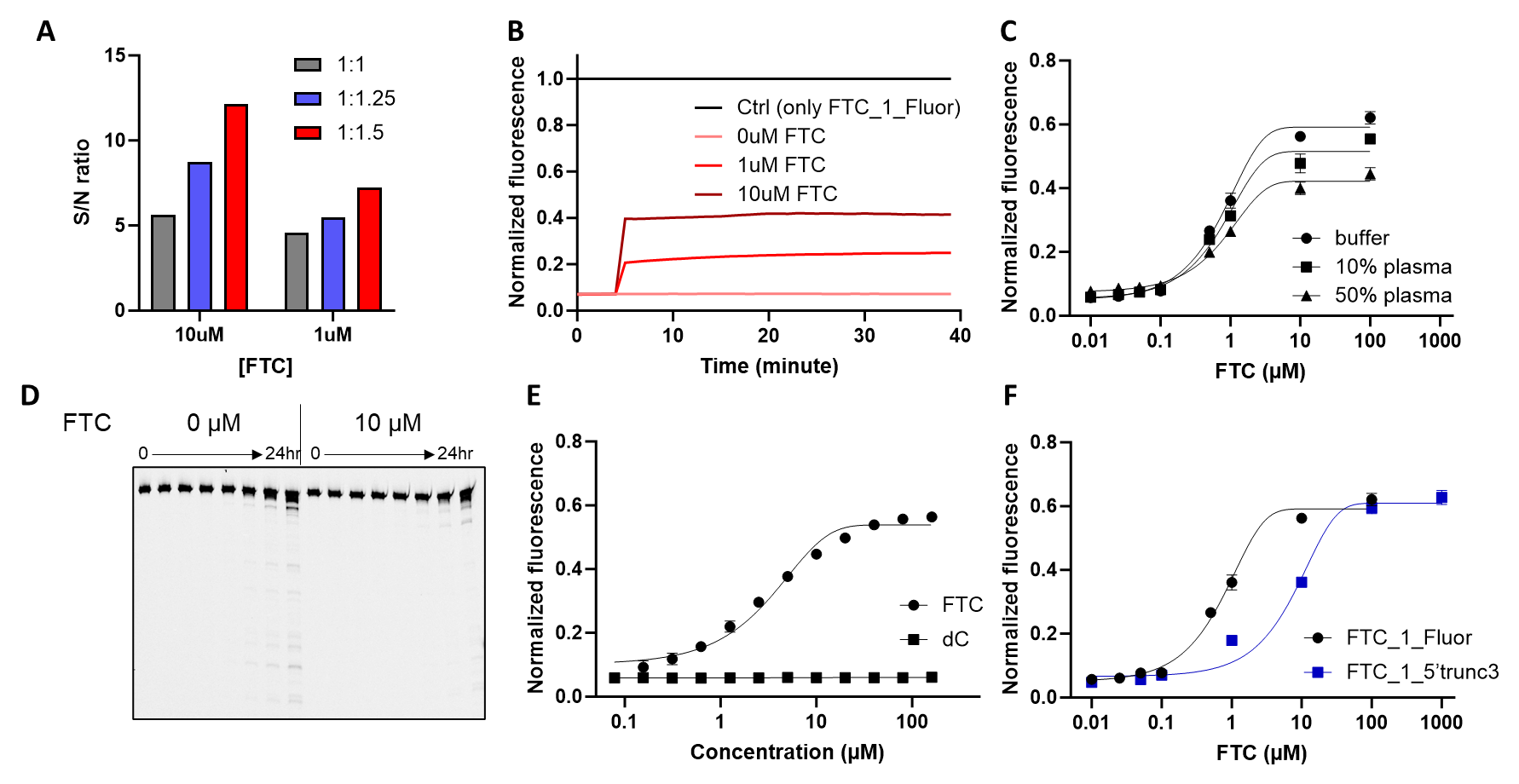 |
| --- |
| Fig S16: Stability in human plasma. The FTC_1_Fluor/Q-Cap_1 probe is stable in 50% human plasma at the time of fluorescence measurement and stability was monitored up to 24 hours, via 20% denaturing polyacrylamide gel. Aliquot was taken at 0, 0.5, 1, 2, 4, 6, 12, 24 hours. |

| 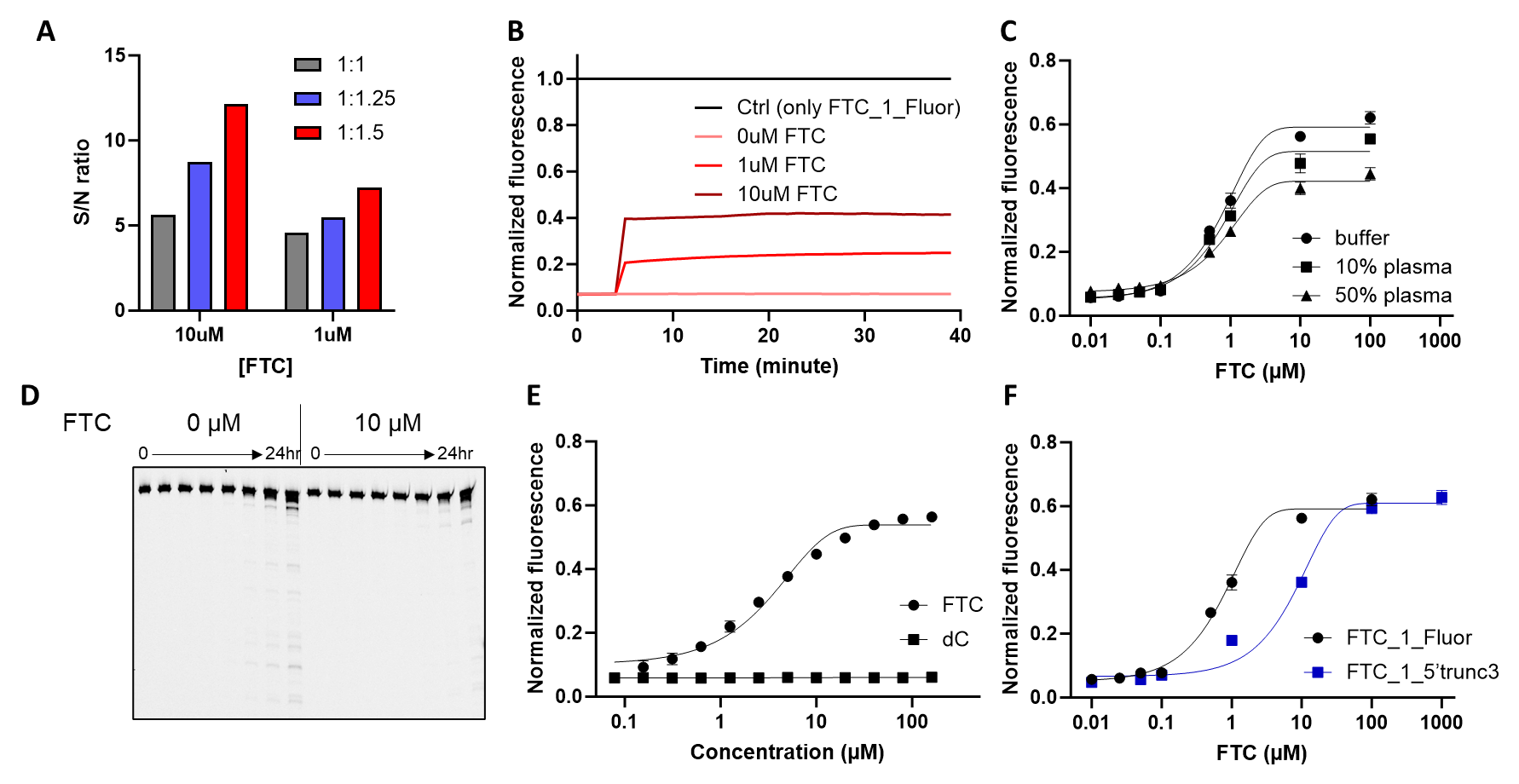 |
| --- |
| Fig S17: Effect of human plasma on sensor performance. The addition of up to 50% human plasma had little effect on the Kd of the sensor. |

|  |
| --- |
| Fig S18: Kinetic analysis of measurement in 50% human plasma. The maximum fluorescent signal was obtained within 1 min following FTC addition (arrow) and maximum fluorescence signal was stable at least 40 minutes. |

| 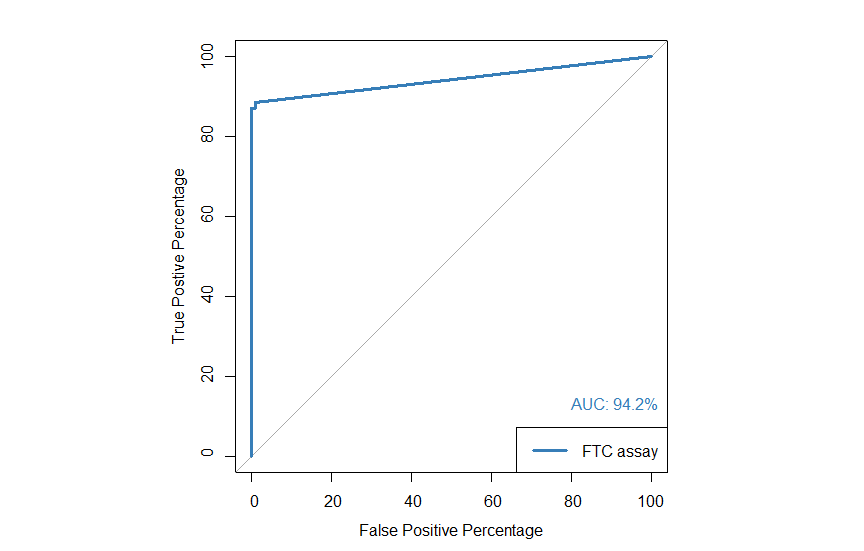 |
| --- |
| Fig S19: Receiver Operating Characteristic curve (ROC curve) of the clinical sample measurement with fluorescence assay. ROC curve was generated using R package PROC. |

| 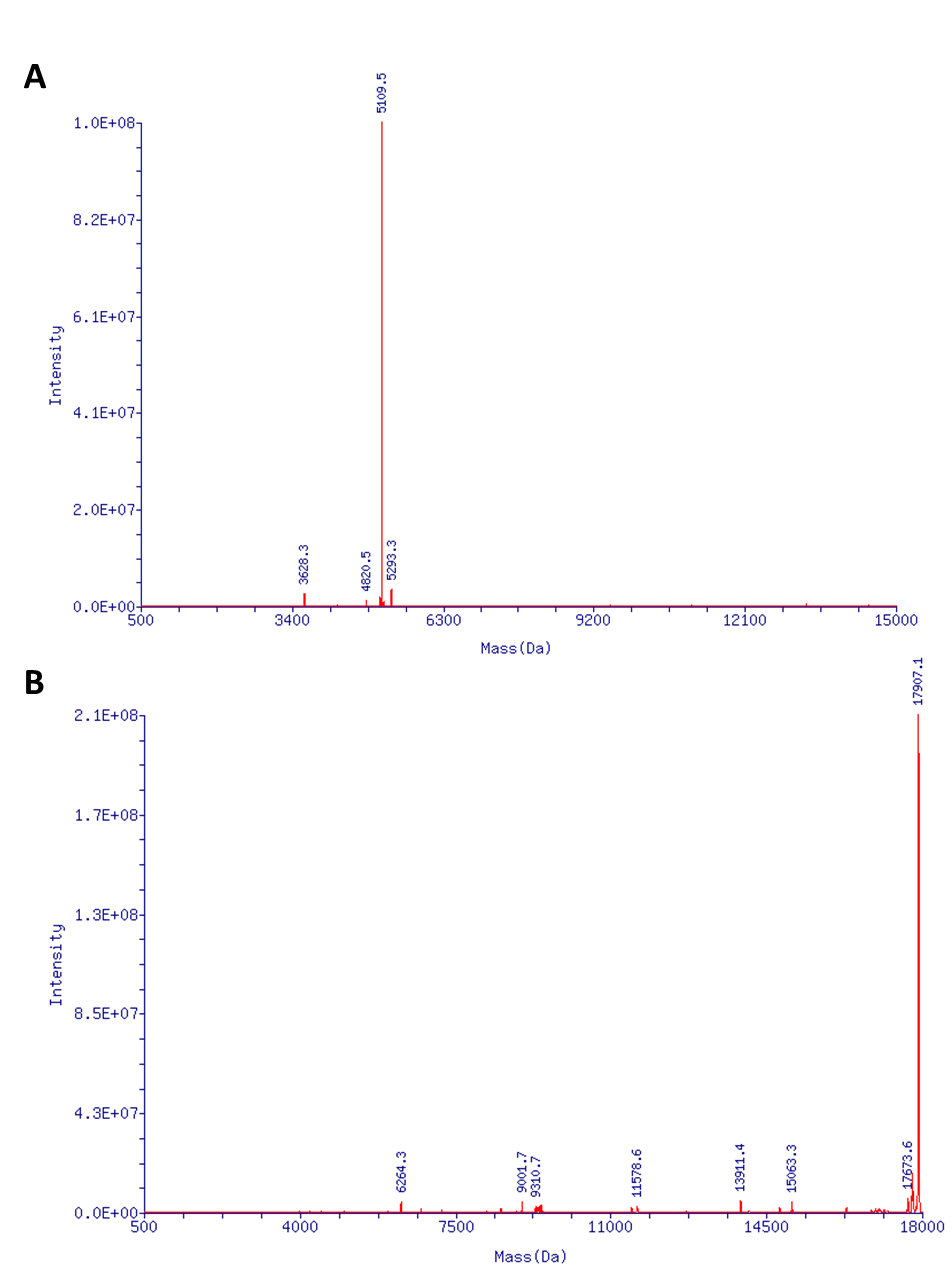 |
| --- |
| Fig S20: ESI-MS spectra of FTC_1_Fluor components. (A) Q-Cap_1. Mass calculated: 5110.5 Da; Mass found: 5109.5 Da. (B) FTC_1_Fluor. Mass calculated: 17908.3 Da; Mass found: 17907.1 Da. |

| 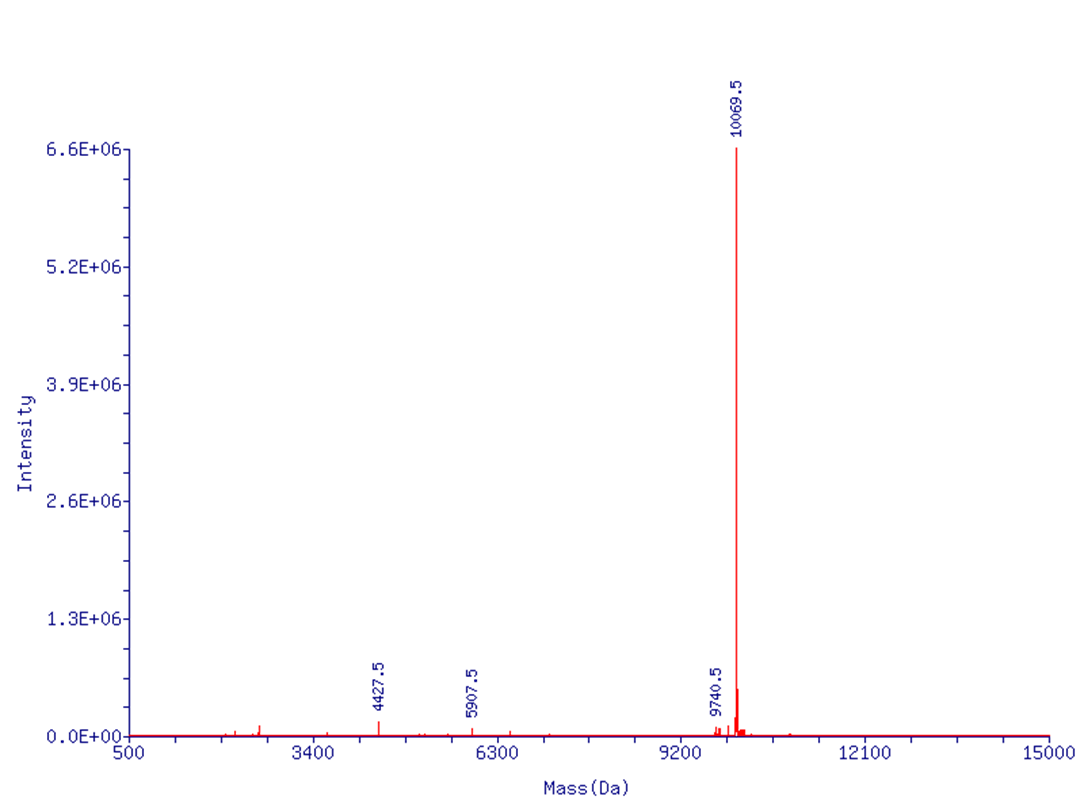 |
| --- |
| Fig S21: ESI-MS spectra of FTC_1_EAB + Surface Modifiers. Mass calculated: 10069.6 Da; Mass found: 10069.5 Da. |
